# RNA-sequencing Analysis of Hybrid Females Reveals a Dominance of Expression of Alleles From Outcrossing Species Over Those From Selfing Species

**DOI:** 10.1093/gbe/evaf098

**Published:** 2025-06-06

**Authors:** Yanwen Shao, Yiwen Zhang, Xiaoliang Ren, Vincy Wing Sze Ho, Yu Bi, Zhongying Zhao, Runsheng Li

**Affiliations:** Department of Infectious Diseases and Public Health, Jockey Club College of Veterinary Medicine and Life Sciences, City University of Hong Kong, Hong Kong, China; Department of Infectious Diseases and Public Health, Jockey Club College of Veterinary Medicine and Life Sciences, City University of Hong Kong, Hong Kong, China; Department of Marine Organism Taxonomy and Phylogeny, Institute of Oceanology, Chinese Academy of Sciences, Qingdao 266071, China; Department of Surgery, The Chinese University of Hong Kong, Hong Kong, China; Department of Infectious Diseases and Public Health, Jockey Club College of Veterinary Medicine and Life Sciences, City University of Hong Kong, Hong Kong, China; Department of Biology, Hong Kong Baptist University, Hong Kong, China; Department of Infectious Diseases and Public Health, Jockey Club College of Veterinary Medicine and Life Sciences, City University of Hong Kong, Hong Kong, China; Department of Precision Diagnostic and Therapeutic Technology, City University of Hong Kong Shenzhen Futian Research Institute, Shenzhen 518057, China; Tung Biomedical Sciences Centre, City University of Hong Kong, Hong Kong, China

**Keywords:** *C. briggsae* and *C. nigoni*, hybrid incompatibility, genetic asymmetry, nuclear–cytoplasmic incompatibility, interspecies

## Abstract

The sister species *Caenorhabditis briggsae* and *Caenorhabditis nigoni* are the first *Caenorhabditis* nematode pair known to produce viable F1 hybrids, making them an ideal model for speciation study. Male F1 hybrids are lethal or sterile depending on the parent of origin, while F1 females, though viable, exhibit distinct phenotypes in fecundity and viability. Besides, both female hybrids could mate with *C. nigoni* males to produce viable progeny but experience hybrid breakdown when crossed with *C. briggsae* males. The molecular mechanisms driving these phenotype variations in F1 females remain unknown. Here, we analyzed the transcriptomes of F1 female hybrids from both crossing directions to examine whether a parent-specific haplotype dominates gene expression in the hybrids and to explore the mechanisms underlying the distinct phenotypes. We showed that in female hybrids from both directions, the *C. nigoni* haplotype was more abundantly expressed and maintained the parental expression patterns better than that of *C. briggsae*. We also observed an upregulation of female-biased genes in the *C. nigoni* haplotype of F1 females, which may explain their compatibility with *C. nigoni* males for producing viable progeny, suggesting a haplotype-specific influence on female reproductive traits. Our mitochondrial gene analysis suggested a nuclear–cytoplasmic incompatibility marked by *cis*-dominated expression patterns of mitochondrial genes, which may contribute to the reduced viability in F1 females. This research provides insights into the expression pattern of interspecies F1 female hybrids and the mechanisms underlying nonlethal hybrid incompatibility defects.

**Significance**

The sister species *Caenorhabditis briggsae* and *Caenorhabditis nigoni* produce viable F1 female hybrids, which exhibit differences in viability between the two crossing directions and variable hybrid incompatibility defects. Our transcriptomic analysis of female parental species and two F1 female hybrids revealed that compared with *C. briggsae* genes, *C. nigoni* genes maintained more consistent expression patterns and dominated in hybrids, particularly influencing female reproduction and oogenic genes. This genetic expression was believed to contribute to the viability of F2 offspring when F1 females mated with *C. nigoni* males. Moreover, our study identified significant nuclear–cytoplasmic incompatibilities marked by *cis*-dominated expression of mitochondrial genes, contributing to reduced viability in one specific crossing direction. Our research sheds light on the genetic mechanisms underlying hybrid viability and will serve as a valuable resource for further research into hybrid incompatibility in nematodes.

## Introduction

*Caenorhabditis* nematodes, characterized by their short lifespans and easily observable phenotypes, serve as excellent models for genetic research. Within this genus, *Caenorhabditis briggsae* and *Caenorhabditis nigoni*, two closely related sister species, are frequently employed to explore reproductive isolation and hybrid incompatibility (HI). The two species are notable for their ability to interbreed bidirectionally, consistently producing fertile female F1 offspring. The F1 female offspring of *C. briggsae* and *C. nigoni* hybrids, though viable and fertile, exhibit sublethal HI defects, leading to F2 breakdown when crossing with *C. briggsae* male (Woodruff et al. 2010; Ting et al. 2014). Additionally, the bidirectional crosses exhibit marked directional characteristics. There is a significant disparity between the viability of the two F1 females, while the F2 progenies are only viable when mated with *C. nigoni*, indicating a directional bias in hybrid fertility.

According to the Darwinian corollary to Haldane's rule, asymmetric inheritance of parental genetic material is a significant contributor to reproductive isolation (Haldane 1922; Cowell 2023). Such asymmetric genetic inheritance is central to the investigation of HI driven by reproductive barriers, particularly concerning mitochondria, chloroplasts, and sex chromosomes (Turelli and Orr 1995; Ellison and Burton 2006; Schilthuizen et al. 2011; Brandvain et al. 2014; Delph and Demuth 2016). In nematode sex chromosome configurations (XX female, XO male), male progenies display pronounced sterility and viability complications (Woodruff et al. 2010; Bi et al. 2015). Our previous research indicated that an interaction between the X chromosome and the autosome was one of the major causes of male sterility in *C. briggsae–C. nigoni* hybrids (Li et al. 2016a). The implications of other asymmetric genetic factors, such as nuclear–cytoplasmic incompatibility, warrant further exploration.

The inheritance, replication, and transcription mechanisms of mitochondrial DNA (mtDNA) have emerged as key areas of research in genetics and metabolism (Anderson et al. 1981; Lemire 2005; Raboin et al. 2010). The maternal inheritance of mtDNA determines the natural conflict between the mitochondrially encoded genome and the nuclear genome in hybrid offspring of two species (Howe et al. 2009; Chang et al. 2015). Nuclear–cytoplasmic incompatibility was reported to cause F2 unviability in two strains of *Caenorhabditis nouraguensis* (Lamelza and Ailion 2017). The results of that study also suggested that nuclear–cytoplasmic conflicts could lead to sublethal HI defects in F1 individuals. Compared with *C. nigoni*, the mtDNA of *C. briggsae* exhibits structural variations, manifested by the insertion of noncoding region (NCR) sequences between *ND3* and *ND5*, as well as high expression of the *ND3* gene (Li et al. 2016b, 2018). Thus, nuclear–cytoplasmic incompatibility may also be a potential factor, causing sublethal defects in F1 females of *C. briggsae* and *C. nigoni*.

Transposable element (TE) reactivation has been implicated in the context of HI (Brideau et al. 2006; Erwin et al. 2015). Abnormal reactivation in TEs has been found in the inviable hybrid males of two *Drosophila* species, but not in the viable intraspecies hybrids (Kelleher et al. 2012; Guerreiro 2014). For nematodes, the activation of TEs also varies between species (Li et al. 2016a). Research on the crossing of male *C. briggsae* and female *C. nigoni* found that different levels of TEs were observed in the two directions of backcrossing F2 hybrids, suggesting that the two species may have distinct TE repertoires and therefore may respond differently to conspecific versus heterospecific TEs (Xie et al. 2022).

Regulatory divergence in hybrids’ gene expression is inherently based on interactions between loci, which play an important role in the process of transcription initiation and establishing certain reproductive barriers. Differences in *cis*-regulatory elements or *trans*-acting factors can result in the misregulation of gene expression in hybrid individuals, leading to developmental defects or physiological disruptions. These regulatory differences can act as barriers to gene flow and contribute to the maintenance of distinct populations or species (Wittkopp 2005; Wray 2007; Wittkopp and Kalay 2011; Gordon and Ruvinsky 2012). Regulatory distribution patterns have been widely used in the study of the differential expression of two parental species and the allele-specific expression (ASE) in F1 hybrids (Merritt et al. 2008; McManus et al. 2010; Sanchez-Ramirez et al. 2021; Viswanath and Cutter 2023). For instance, Sanchez-Ramirez et al. (2021) specifically examined *C. briggsae* × *C. nigoni* hybrids, comparing female and male offspring to elucidate sex-specific differences in regulatory and expression profiles. Moreover, many other studies in flies (Wittkopp et al. 2004; Wang et al. 2016), yeast (Tirosh et al. 2009; Emerson et al. 2010), and plants (Li et al. 2022; Wan et al. 2022) report that expression divergence of reciprocal F1 transcriptomes is highly correlated with intrinsic regulatory differences (*cis/trans*).

Based on the phenotypic and genetic asymmetry within interspecific hybrids and the current understanding of evolutionary theory, we examined the transcriptomes of fertile hybrid female offspring of crosses between *C. briggsae* and *C. nigoni*, aiming to reveal the characteristics of and differences between F1 female hybrids through RNA-seq analysis of F1 hybrids and parental species. We also provided explanations for the essential phenotypes of nonlethal HI in several respects, including cytoplasmic–nuclear comparison, sex-biased tendency, inheritance, and regulatory profiles.

## Results

### Reduced Haplotype Expression Variability was shown in F1 Hybrids

Stranded RNA-seq data were generated using young adult female worms from wild-type *C. briggsae* (strain AF16), *C. nigoni* (strain JU1421), and F1 hybrids crossed in two directions. BN refers to the F1 female hybrid from the crossing of *C. briggsae* males and virgin *C. nigoni* females; NB is the F1 female hybrid from the crossing of *C. nigoni* males and sperm-depleted *C. briggsae* hermaphrodites. Sequencing was carried out in 2 × 150 bp mode using an Illumina MiSeq. The *C. nigoni* samples were treated in triplicate, while the other samples were treated in duplicate (supplementary table S1, Supplementary Material online).

**Fig. 1.**
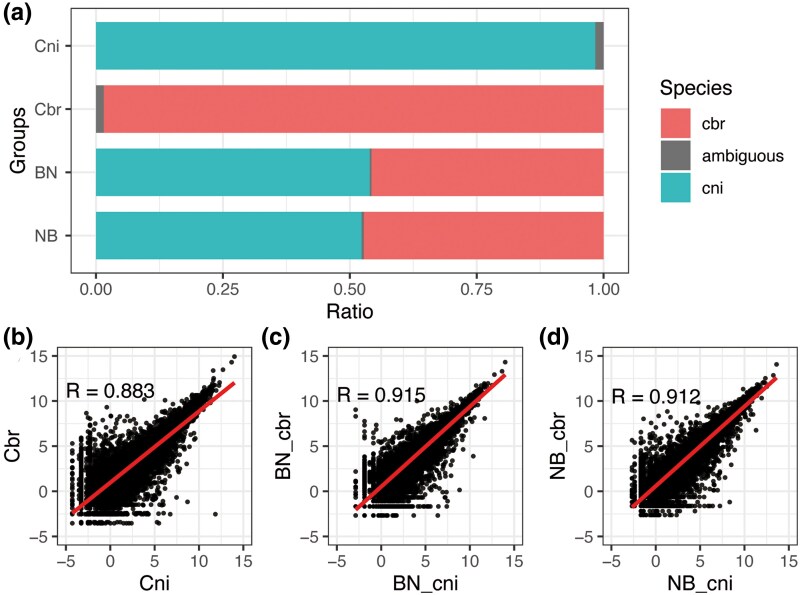
Reduced gene expression variability in F1 female hybrids. The gene expression differences between haplotypes in the F1 hybrids were partially reduced. a) RNA-seq reads mapping ambiguity is shown against a combined reference of both *C. briggsae* and *C. nigoni* genomes. The data represent an average of multiple replicates: three for the *C. nigoni* group (Cni) and two for the *C. briggsae* group (Cbr), as well as for the hybrid groups (BN and NB). b to d) Spearman correlation of one-to-one ortholog gene expression between wild-type *C. nigoni* and *C. briggsae* young adult females (b), and between the ortholog gene expression from *C. nigoni* and *C. briggsae* haplotypes in the BN (c) and NB (d) worms. Cni, *C. nigoni*; Cbr, *C. briggsae*; BN, the F1 female hybrid from the crossing of *C. briggsae* males and virgin *C. nigoni* females; NB, the F1 female hybrid from the crossing of *C. nigoni* males and sperm-depleted *C. briggsae* hermaphrodites; BN_cni, the *C. nigoni* haplotype in the young adult BN; BN_cbr, the *C. briggsae* haplotype in BN; NB_cni, the *C. nigoni* haplotype in the young adult NB; NB_cbr, the *C. briggsae* haplotype in NB.

The next-generation sequencing (NGS) reads were mapped against the combined reference genomes from both *C. briggsae* and *C. nigoni* (see Methods for details). However, as sister species, *C. briggsae* and *C. nigoni* had an average corresponding coding sequence similarity of over 92% (Li et al. 2016a). One NGS read could sometimes not be assigned to only a single species because the mapped region was almost identical in both genomes. To exclude the mapping bias in hybrids, we simulated the mapping scenario by combining RNA reads from two parent species in equal proportions within FASTQ data and aligning them to the combined genome of both species (supplementary fig. S1, Supplementary Material online). The results revealed a balanced 50:50 mapping ratio between the two species, with no observed bias toward *C. nigoni*. And to verify the accuracy of mapping NGS reads to the combined genome, we analyzed the mapping of each read individually, labeling any read mapped to both genomes as ambiguous (Fig. 1a). When using the combined genome as a reference, <2% of RNA-seq reads from the two parental female species were classified as ambiguous, and this rate was no more than 0.5% for the F1 hybrid data.

In the BN hybrids, 54% of the RNA-seq reads derived from the *C. nigoni* haplotype, with the rest from the *C. briggsae* haplotype. Likewise, 53% of the RNA-seq reads were derived from the *C. nigoni* haplotype in NB hybrids. The dominance of reads derived from the *C. nigoni* haplotype in hybrids may be attributed to its larger genome size and increased number of coding genes. Since F1 female hybrids inherit half of their genome from each parental species, the higher transcriptome complexity of *C. nigoni* provides a natural bias toward higher expression from this haplotype.

The gene expression differences between haplotypes in the F1 hybrids were partially reduced relative to their parental species (Fig. 1b to d). A total of 16,269 pairs of one-to-one orthologs between *C. briggsae* and *C. nigoni* were defined using mutual Blastp (see Methods for details) for further expression analysis. We performed the haplotype differential gene analysis based on the one-to-one orthologs. For comparisons between parental species, genes were compared directly to their corresponding one-to-one orthologs. Similarly, for allele-specific comparisons between haplotypes in hybrids, genes from one haplotype were compared with their orthologous alleles in the other haplotype. First, the correlation analysis at the allele level showed that the young adult females of wild-type *C. nigoni* and *C. briggsae* had a Spearman correlation coefficient (*R* value) of 0.883 (Fig. 1b), and the correlation between the two haplotypes in both F1 hybrids was even greater, with BN having an *R* value of 0.915 and NB having an *R* value of 0.912 (Fig. 1c and d). Second, in the comparison between the two parental species, 2,352 of the 10,381 expressed one-to-one ortholog pairs (23%) demonstrated a significant change (false discovery rate [FDR] < 0.05 and |log_2_ fold change| > 1; supplementary fig. S2a, Supplementary Material online). When analyzing the gene expression between different haplotypes within the two F1 hybrids, a smaller number of differentially expressed ortholog pairs were identified; 1,343 and 1,811 pairs were found in the BN and NB hybrids, respectively (FDR < 0.05 and |log_2_ fold change| > 1; supplementary fig. S2b and c, Supplementary Material online). The overall correlation was higher in F1 hybrids, and fewer genes showed significant ASE differences compared with DEG analysis using parental species. The ASE differences in hybrids were less common than differential gene expression between the parental species.

### Inheritance Pattern Analysis showed that *C. nigoni* Genes Maintain Their Expression Pattern Better than *C. briggsae* Genes in F1 Female Hybrids

**Fig. 2.**
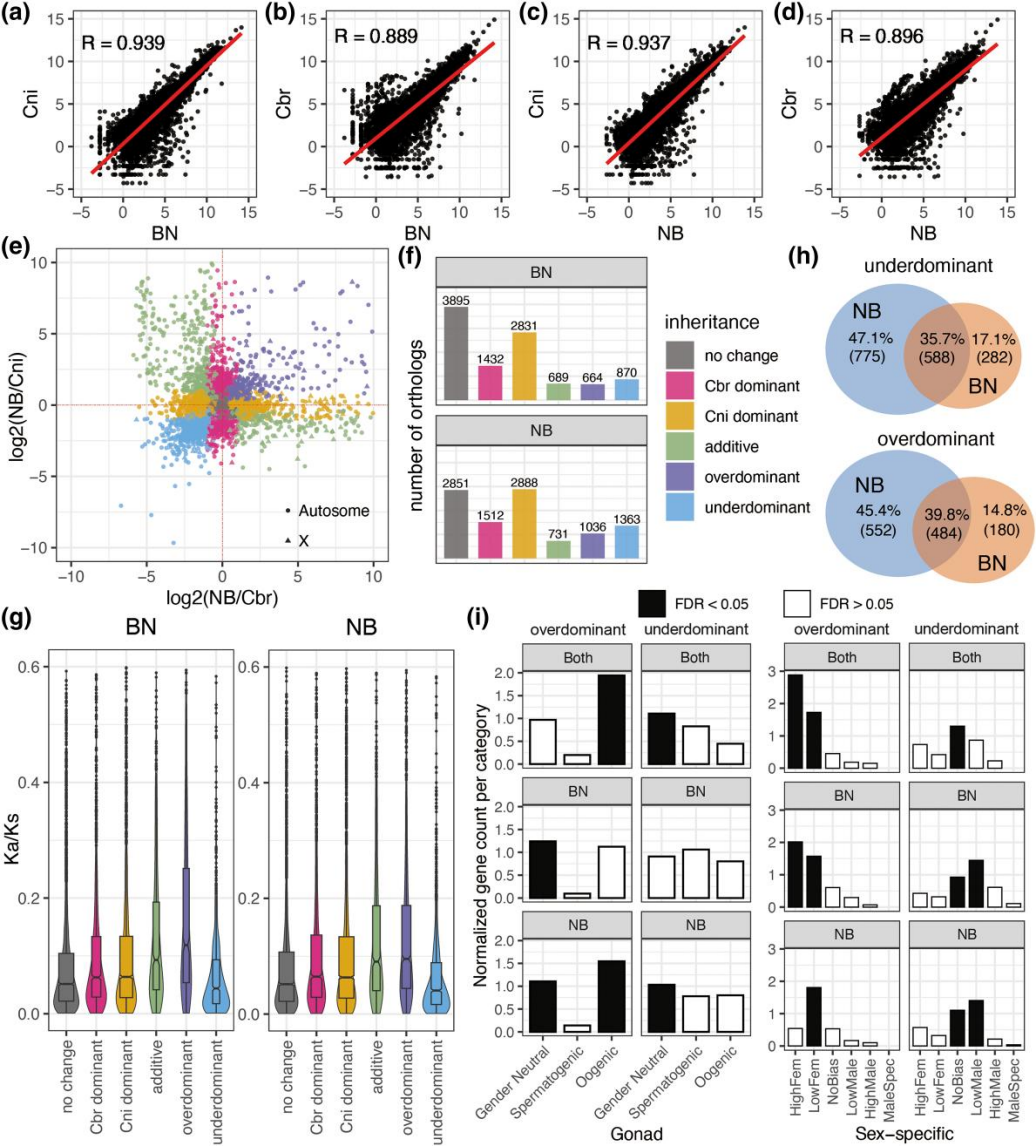
Comparative analysis of gene expression and inheritance patterns in F1 hybrids and wild-type *C. nigoni* and *C. briggsae*. a to d) Spearman correlation of all the one-to-one ortholog gene expressions in F1 hybrids and in wild-type young adult female worms: Spearman correlation of ortholog gene expression between wild-type *C. nigoni* and its haplotype in BN (a) and NB (b); Spearman correlation of ortholog gene expression between wild-type *C. briggsae* and its haplotype in BN (c) and NB (d). e and f) Expression inheritance modes between two species in NB. e) The dot plot shows the log_2_ expression differences between NB hybrids and each parent species. Orthologs were classified into patterns of no change, Cbr dominant, Cni dominant, additive, overdominant, and underdominant. (f) The number of inheritance profiles of all expressed orthologs in the two F1 hybrids. g) Protein sequence divergence (*K*_a_/*K*_s_) of the two F1 hybrids according to the inheritance profiles in BN and NB, i.e. the ratio of the number of nonsynonymous substitutions per nonsynonymous site (*K*_a_) to the number of synonymous substitutions per synonymous site (*K*_s_). h) Venn plots of transgressive genes shared by the two F1 hybrids. i) Enrichment analysis against gonad-related genes and sex-specific gene categories of shared overdominant and underdominant regulated genes in the two F1 hybrids (significance cutoff is FDR = 0.05). The “Normalized gene count per category” was obtained by the count number of each gene set divided by the background number of the sex category.

Compared with the *C. briggsae* haplotype, the expression of the *C. nigoni* haplotype in both hybrids (Fig. 2a and c) tended to have a stronger correlation with that of its parental species (Fig. 2b and d). To further compare the expression patterns of the F1 female hybrids, we examined the inheritance patterns of each orthologous pair by comparing the gene expression in the two F1 hybrids with that in the parental species. Based on the inheritance pattern of gene expression, all 10,381 expressed one-to-one orthologs could be classified into the following previously described categories (McManus et al. 2010; Sanchez-Ramirez et al. 2021): additive, dominant (*C. briggsae* or *C. nigoni*), transgressive (overdominant and underdominant), and no change (Fig. 2e, supplementary fig. S3a, Supplementary Material online).

The number of *C. nigoni*–dominant orthologs was much higher than that of *C. briggsae*–dominant orthologs in both F1 hybrids. Among the 10,381 expressed ortholog pairs, the proportions of genes assigned to the various categories showed similar distributions in both F1 hybrids (Fig. 2f). The proportion of *C. nigoni*–dominant ortholog pairs was almost twice that of *C. briggsae*–dominant ortholog pairs in both hybrids, with 2,831 (27.3%) and 2,888 (27.8%) *C. nigoni*–dominant orthologs in BN and NB, and 1,432 (13.8%) and 1,512 (14.6%) *C. briggsae*–dominant orthologs in BN and NB (Fig. 2g), respectively.

Additionally, we calculated the synonymous-to-nonsynonymous ratio per nucleotide site (*K*_a_/*K*_s_) for the genes associated with various inheritance patterns (Fig. 2g). The genes of overdominant and additive patterns showed higher *K*_a_/*K*_s_ ratios than those of no-change and underdominant patterns. We also observed that the *K*_a_/*K*_s_ ratios for *C. briggsae*– and *C. nigoni*–dominant orthologs were similar.

In summary, we found that for the orthologs between the two parental species, those expressed from the *C. nigoni* alleles were more likely to maintain their parental expression pattern in F1 hybrids than their *C. briggsae* counterparts. Both viable F1 hybrids exhibited a more similar gene expression pattern to that of *C. nigoni* than to that of *C. briggsae* in correlation and inheritance expression patterns.

### NB had More Transgressively Expressed Genes than BN, and Overdominant Genes were Female Biased

The F1 hybrids had phenotypic asymmetry in various directions (Woodruff et al. 2010). For example, female BN exhibited a higher fecundity and viability than female NB (456 females vs. 53 females, 45% viable vs. 28% viable; Woodruff et al. 2010). Transgressive genes (both underdominant and overdominant), which indicate gene misexpression that exceeds the range of both parents, are likely to be associated with hybrid dysfunction. To assess the haplotype expression asymmetry in BN and NB hybrids, we compared the expression inheritance profiles between the two F1 hybrids in different crossing directions.

NB had 23.1% transgressive genes (1,036 overdominant and 1,363 underdominant), compared with only 14.8% in BN (664 overdominant and 870 underdominant), which was consistent with the lower viability of NB.

We next explored the differences in transgressive orthologs between BN and NB (Fig. 2h). Between the two F1 hybrids, there were a total of 1,645 pairs of underdominant orthologs, of which only 588 (35.7%) were shared, while 775 (47.1%) were unique to NB. Similarly, among the 1,216 pairs of overdominant orthologs, 484 (39.8%) were shared between BN and NB, while 552 (45.4%) exhibited overdominance only in NB.

We further investigated the enrichment of gonad and sex-specific categories (see Methods for details) within gene sets associated with classified transgressive genes (Fig. 2i, supplementary fig. S9a, Supplementary Material online). We found a significant enrichment of the overdominant genes in oogenic and female-biased terms, whereas underdominant genes predominantly aligned with male-biased or hermaphrodite terms.

Both gene ontology (GO) and Kyoto Encyclopedia of Genes and Genomes (KEGG) pathway enrichment analysis of these transgression-related gene sets revealed that the underdominant gene set shared by BN and NB was enriched in pathways related to TCA oxidoreductase activity (supplementary fig. S4a and b, Supplementary Material online). The set of genes that were only underdominant in NB also showed enrichment in KEGG pathways related to mitochondrial functions.

To conclude, NB had more transgressive orthologs than BN, which may potentially lead to lower viability in NB females than BN females. Additionally, the enriched GO pathways suggest a role of mitochondrial misregulation in the reduced viability of NB females.

### More Orthologs in the F1 Female Hybrids showed *cis* than *trans* Effects in Regulatory Profiles

**Fig. 3.**
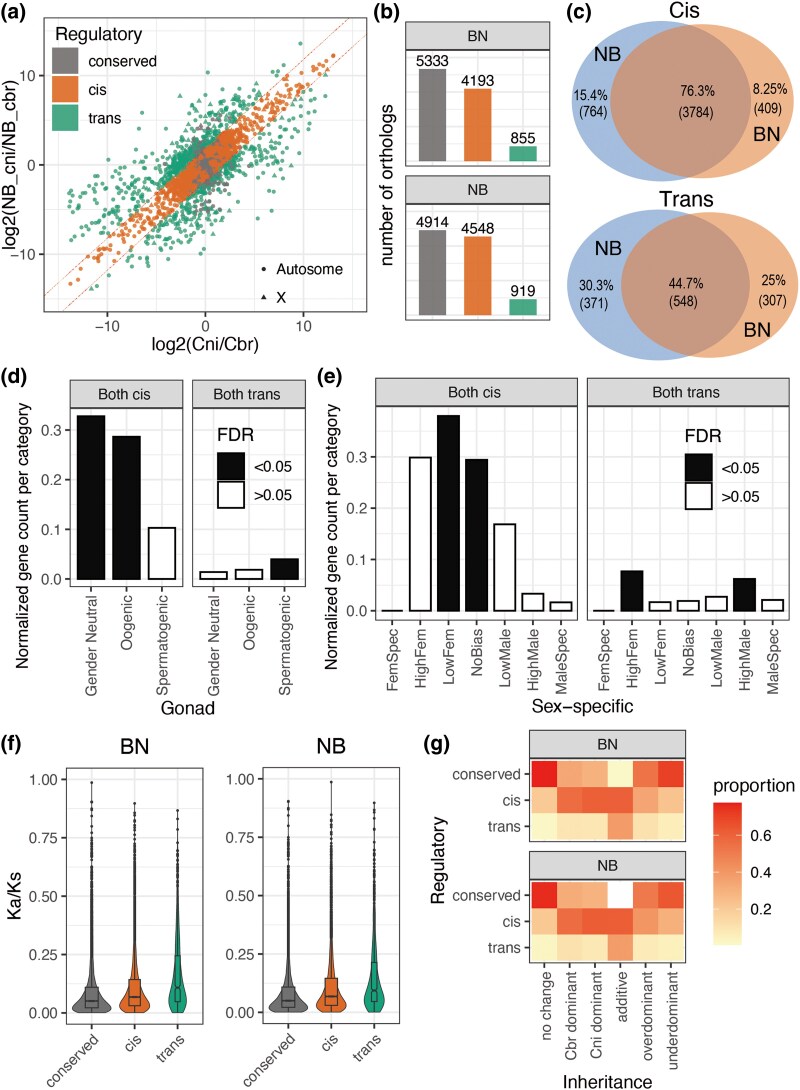
Regulatory profiles of the two F1 hybrids. a) The dot plot shows expression divergence between species (*x*-axis) and ASE in the NB hybrid (*y*-axis), demonstrating the extent of *cis-*effects on allele expression. Orthologs were defined as conserved, *cis*, and *trans*. b) The numbers of regulatory profiles of all expressed orthologs in the two F1 hybrids. c) Venn plots of *cis-* and *trans-*regulated genes shared by the two F1 hybrids. d, e) Enrichment analysis against gonad-related genes (d) and sex-specific gene categories (e) of shared *cis*- and *trans*-regulated genes in the two F1 hybrids (significance cutoff is FDR = 0.05). The “Normalized gene count per category” was obtained by the count number of each gene set divided by the background number of the sex category. f) Protein sequence divergence (*K*_a_/*K*_s_) of the two F1 hybrids according to regulatory categories, i.e. the ratio of the number of nonsynonymous substitutions per nonsynonymous site (*K*_a_) to the number of synonymous substitutions per synonymous site (*K*_s_). g) Heat map showing the proportion of orthologs within each inheritance category classified into three different regulatory effects (*cis*, *trans*, or conserved). The proportion was calculated by the number of shared regulatory and inheritance categories dividing the overall number of the corresponding inheritance category.

We compared the expression between the parental species and the ASE in the two F1 hybrids to identify the regulatory patterns. We classified the genes into three classes: *cis*, *trans*, and conserved (Fig. 3a, supplementary fig. S3d, Supplementary Material online). Conserved genes showed no significant differences in expression either between parental species or between Cni and Cbr alleles within the F1 hybrids. The definition of *cis* and *trans* effects followed the traditional framework described by Wittkopp et al. (2004). *Cis* effects in F1 hybrids indicated that the allele expression mimicked the expression pattern from the parental species, whereas *trans* effects manifested as expression patterns that differed from both parents, suggesting influences from the *trans* environment in the F1 hybrid. Thus, genes classified as *trans* are not only inferred to have *trans* effects but could also exhibit *cis* effects in many cases.

In the BN hybrid, the conserved genes predominated (5,333, 51.4%), followed by *cis* (4,193, 40.4%) and *trans* (855, 8.2%) effects (Fig. 3b). The NB hybrid showed a similar trend but with a higher proportion of nonconserved ortholog pairs than BN, with larger percentages of *cis* (4,193, 43.8%) and *trans* (919, 8.9%) genes.

In the regulatory profiles of BN and NB, 3,784 of the ortholog pairs showed consistent *cis*-regulated expression, and 548 were consistently *trans* regulated (Fig. 3c). Our analysis of sex-related terms revealed a significant enrichment of gender neutral and oogenic genes in the *cis*-regulated gene set, while spermatogenic genes were significantly enriched in the *trans*-regulated gene set (Fig. 3d). Similarly, *cis*-regulated genes were mainly concentrated in female-biased gene terms, while *trans*-regulated genes were also enriched in male-biased or hermaphrodite terms (Fig. 3e, supplementary fig. S9b, Supplementary Material online).

The genes of *cis* and *trans* effects tended to regulate different pathways in the F1 hybrids in the analyses of both GO (supplementary fig. S5a, Supplementary Material online) and KEGG (supplementary fig. S5b, Supplementary Material online) pathways. The shared *cis* genes were more enriched in DNA and RNA production–related pathways (e.g. DNA repair, spliceosome), while the *trans* genes were more enriched in downstream pathways (e.g. protein phosphatase, longevity).

In the two F1 hybrids, *trans*-regulated genes showed the highest *K*_a_/*K*_s_ values, followed by *cis*-regulated genes, and finally conserved genes (Fig. 3f).

After obtaining the regulatory and inheritance profiles of the two F1 hybrids and their parental species, we generated a heat map based on the proportions of different regulatory effects within each inheritance category (Fig. 3g). The distributions of NB and BN were very similar. Additionally, the additive category had the highest proportion of *trans-*regulated genes.

### Chromosomal Distribution of Inheritance Patterns showed X-biased Transgressive Gene Enrichment

Based on the expression inheritance and regulatory profiles, we summarized the distributions of these categories across different chromosomes (supplementary fig. S3b and e, Supplementary Material online) and conducted an enrichment analysis of the distributions (Fisher's exact test; patterns with *P* < 0.01 and |log_2_ odds ratio| > 0.4 were considered to be significantly changed; supplementary fig. S3c and f, Supplementary Material online).

The inheritance profile analysis revealed that the X chromosome was enriched in underdominant genes and depleted of overdominant genes compared with autosomes (supplementary fig. S3b and c, Supplementary Material online). This was especially evident in NB, with 414 (25.8%) underdominant orthologs in the X chromosome (Fisher's exact test, log_2_ odds ratio = 1.52, *P* < 2*e*^−51^). In addition, there were 245 (15.3%) underdominant orthologs in BN (Fisher's exact test, log_2_ odds ratio = 1.23, *P* < 1.3*e*^−23^).

Regarding the regulatory pattern, the distribution of *trans*-regulatory genes showed considerable variations across different chromosomes in both F1 hybrids. Chromosome V featured the highest number of *trans*-regulatory genes, demonstrating significant enrichment (Fisher's exact test, log_2_ odds ratio = 0.8, *P* < 1*e*^−11^), followed by the X chromosome, although the difference was not significant. Moreover, the X chromosome presented the lowest number of *cis*-regulated genes, indicating a significant depletion (Fisher's exact test, log_2_ odds ratio < −0.6, *P* < 2*e*^−14^).

In summary, the chromosome distributions implied that the misregulation of the inheritance mode in the X chromosome may play a significant role in the observed HI in F1 hybrids, especially in NB.

### The Overrepresentation of *cis*-Regulated Mitochondrial Genes Suggests a Nuclear–Cytoplasmic Incompatibility

**Fig. 4.**
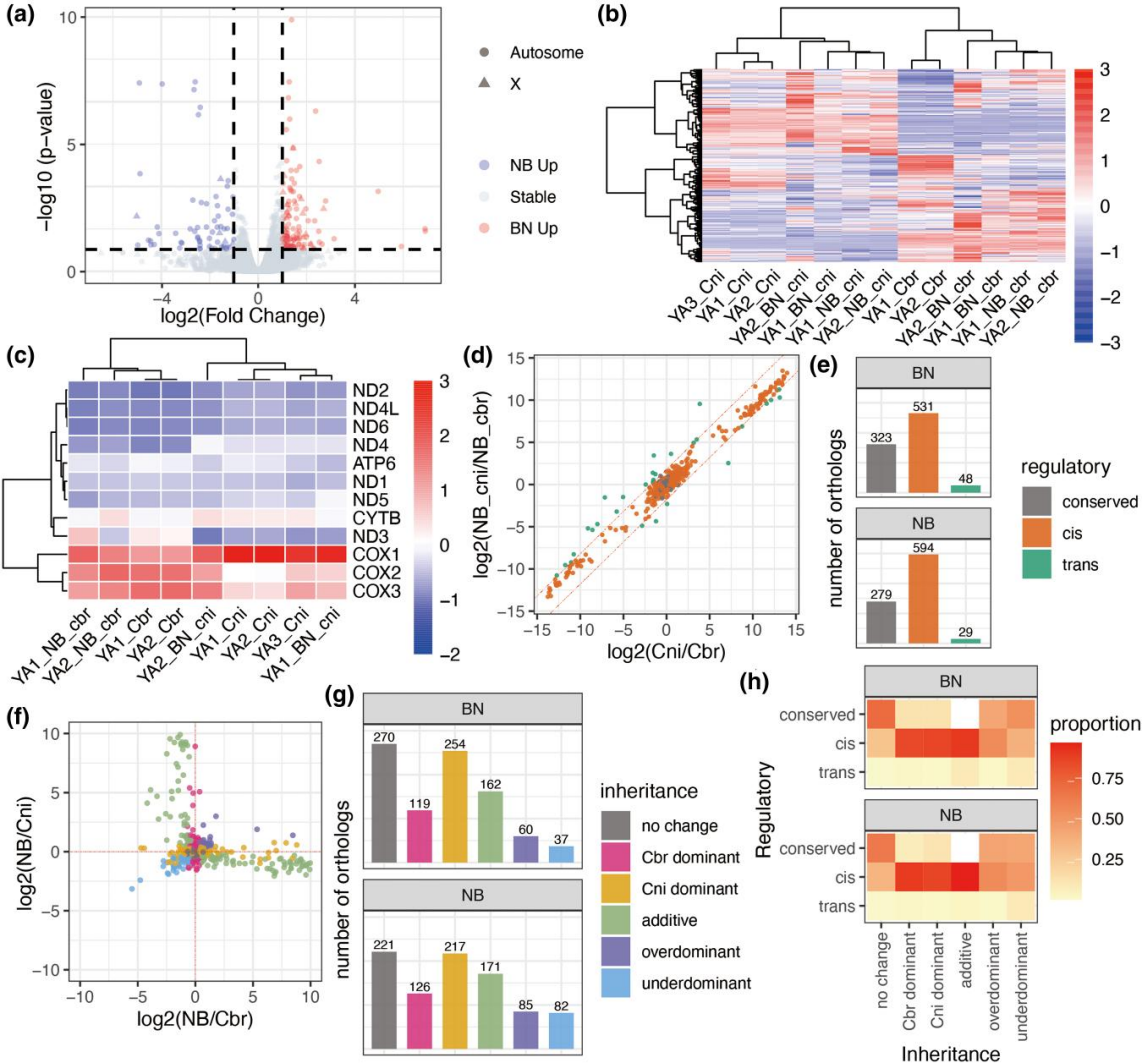
Analysis of mitochondrial genes of two F1 hybrids. a) The volcano plot shows the overall differential expression genes between BN and NB. b) Heat map showing the expression trend of all one-to-*n* nuclear-encoded mitochondria-related genes in the CPM level of two species’ haplotypes. c) The heat map shows the expression levels of 12 mitochondria-encoded genes. d, e) The *cis-* and *trans-*regulated genes in 902 expressed one-to-*n* orthologs of mitochondrial-related genes of NB. The dot plot shows the method used to define conserved, *cis*, and *trans* genes in the F1 hybrids d) and the numbers of regulatory profiles of mitochondria-related genes in the two F1 hybrids (e). f, g) Expression inheritance modes between two species in one-to-*n* orthologs of mitochondria-related genes of NB. The dot plot shows the method used to classify the one-to-*n* orthologs into expression inheritance categories (f) and the numbers of inheritance categories of mitochondria-related genes of the two F1 hybrids (g). h) Heat map showing the proportions of different regulatory effects within each inheritance category for mitochondria-related genes. The proportion was calculated by the number of shared regulatory and inheritance categories dividing the overall number of the corresponding inheritance category.

When exploring the DEGs and overlapping transgressive orthologs between BN and NB, we found that several GO pathways involving oxidation–reduction reactions were enriched in the gene set with higher expression in NB (Fig. 4a, supplementary fig. S4a, Supplementary Material online). We therefore further explored the impact of mitochondrial genes on the HI of F1 hybrids. Due to the maternal inheritance and the unique transcriptional mechanism of mtDNA, nuclear–cytoplasmic incompatibility is also a crucial factor in genetic asymmetry and HI. Both mitochondria-encoded and nucleus-encoded mitochondrial genes were used to further investigate whether genes highly associated with mitochondria in F1 hybrids exhibited a distinct expression pattern compared with that of overall gene expression. Nucleus-encoded mitochondrial genes in *C. briggsae* and *C. nigoni* were defined by identifying human orthologs (see Methods for details; supplementary table S5, Supplementary Material online).

The genes encoded by the Cbr haplotype and the Cni haplotype were grouped together, with a pronounced *cis*-regulatory effect observed for the nucleus-encoded mitochondrial (Fig. 4b) and mitochondria-encoded genes (Fig. 4c, supplementary fig. S6b, Supplementary Material online). Besides, expression of mitochondrial-encoded genes with Cbr signature was observed only in NB, as evidenced by the NCR next to *ND3* and *CYTB* (supplementary fig. S7, Supplementary Material online). The regulatory profiles of nucleus-encoded mitochondrial genes showed the same pattern as that of the orthologs overall, which highlights the predominance of *cis*-regulated genes, as found in both NB and BN (Fig. 4d and e, supplementary fig. S6c and d, Supplementary Material online). The proportion of *cis*-regulated genes exceeded that of overall orthologs (Fig. 4e and b).

Most mitochondria- and nucleus-encoded mitochondrial genes displayed Cni dominant inheritance patterns in both F1 hybrids, followed by no-change and additive patterns (Fig. 4f and g). After analyzing the distribution of mitochondria-related genes across inheritance and regulatory categories, we observed that *cis*-regulated genes dominated in both additive and dominant gene sets, contributing to over 85% of these categories (Fig. 4h, Fisher's exact test, log_2_ odds ratio > 0.6, *P* < 1*e*^−8^). Conversely, the proportions of *cis*-regulated elements in transgressive genes were significantly lower than the overall proportion of *cis*-regulated genes (Fig. 4h, Fisher's exact test, log_2_ odds ratio < −1.5, *P* < 1*e*^−40^).

Generally, the genetic asymmetry caused by maternally inherited mitochondria is reflected in a much higher proportion of *cis*-effect genes among mitochondrial genes than among the overall genes. Additionally, the increased dysfunctions in NB manifested as reduced fecundity and viability compared with BN may be potentially linked to the enrichment of DEGs related to mitochondrial pathways.

### More Maternally Imprinted Genes than Paternally Imprinted Genes were Observed

**Fig. 5.**
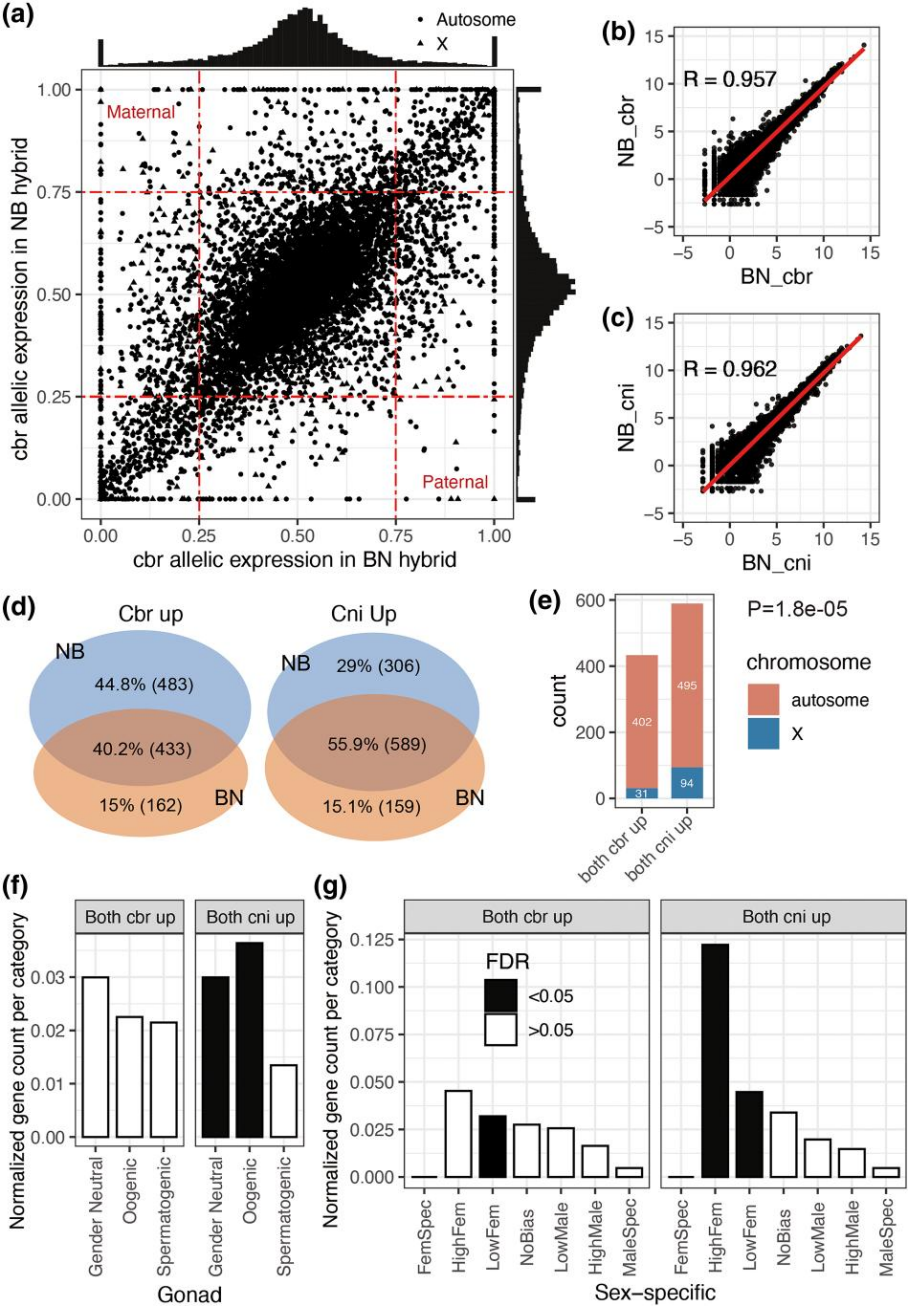
Comparison between two haplotypes of the two F1 hybrids. a) The paternal and maternal imprinted genes in the F1 hybrids. The *x* and *y* axes show the fraction of *C. briggsae* allelic expression in the paternal (BN) and maternal (NB) directions, respectively. The fraction of *C. nigoni* in the paternal (NB) or maternal (BN) direction is thus a fraction equal to (1 − cbr). Histograms of the summed frequency of the *C. briggsae* allelic expression are shown on the top and the right side for BN and NB, respectively. b and c) Spearman correlation of all the one-to-one ortholog gene expressions between BN and NB for the *C. briggsae* (b) and *C. nigoni* (c) haplotypes. d) Venn plots showing the numbers of differentially expressed alleles and shared orthologs between the two F1 hybrids. To obtain differentially expressed alleles, the expression of BN_cbr and BN_cni and that of NB_cbr and NB_cni were compared, then in the comparison of two F1 hybrids with different alleles, the orthologs upregulated by Cbr were classified as “Cbr up” and those upregulated by Cni were classified as “Cni up,” while “Cbr up” orthologs shared by the two F1 hybrids were classified as “both cbr up” and “Cni up” orthologs shared by the two F1 hybrids were classified as “both cni up.” e) Bar plot displaying autosomes and X chromosome distribution based on shared orthologs obtained from (d). The *P*-value of Fisher's exact test (*P* < 1.8*e*^−05^) indicates that the number of genes from the X chromosome in the “both cni up” group is significantly higher than that in the “both cbr up” group. f, g) Enrichment analysis of shared orthologs from (d) against the gonad-related genes (f) and sex-specific genes categories (g) (significance cutoff is FDR = 0.05). The “Normalized gene count per category” was obtained by the count number of each gene set divided by the background number of the sex category.

Analyzing the F1 hybrids resulting from reciprocal crosses, we examined gene imprinted by assessing the expression ratios of alleles from the two parental species for all expressing orthologs. The plot in Fig. 5a displays the fraction of *C. briggsae* allelic expression in the paternal (BN) and maternal (NB) directions. Overall, the majority of orthologs showed a slight maternal tendency regarding the two parental species, with 5,236 maternal and 4,920 paternal orthologs.

The upper left and lower right corners of the imprinted genes represent the maternally and paternally imprinted genes (Fig. 5a), respectively. In total, there were 45 maternally imprinted and 15 paternal imprinted genes (supplementary tables S7 and S8, Supplementary Material online). Genes from the X chromosome accounted for the highest number of imprinted genes (supplementary fig. S8a, Supplementary Material online).

Six maternal and two paternal imprinted genes were confirmed in previous reports on imprinted genes (Skaar et al. 2012), due to their high protein similarity to genes from other species. GO and KEGG analysis showed that the maternal imprinted genes were enriched in cuticles and transporters (supplementary fig. S8b and c, Supplementary Material online).

### The Upregulated *C. nigoni* Alleles in both F1 Hybrids were Enriched on the X Chromosome and Female-biased Terms

Given the high correlation within the same haplotypes between both BN and NB (Fig. 1c and d) and the minimal DEGs between BN and NB (supplementary fig. S1d, Supplementary Material online), we further analyzed the differentially expressed alleles between the two haplotypes of F1 hybrids (*P* < 0.05, |log_2_ fold change| > 1, supplementary figs. S2b, c, and S8d, Supplementary Material online). We identified 589 pairs of orthologs with consistently higher expression of *C. nigoni* alleles than *C. briggsae* in both BN and NB F1 hybrids, and 433 pairs where *C. nigoni* alleles were consistently less expressed in both hybrids (Fig. 5d).

In *C. nigoni*, 94 out of 495 orthologs that were upregulated (19%) belonged to the X chromosome, with a significance of *P* = 1.8*e*^−5^. In contrast, in *C. briggsae*, only 31 out of 402 upregulated orthologs (7.7%) were associated with the X chromosome (Fig. 5e). Furthermore, we investigated whether divergence genes common to the two F1 hybrids were enriched in sex-related categories. Significant enrichment of oogenic and high female-biased genes was observed only in the orthologs with upregulated expression of *C. nigoni* alleles compared with *C. briggsae* alleles (FDR < 0.05, Fig. 5f and g, supplementary fig. S9c, Supplementary Material online), while only a low female enrichment was shown in the orthologs with upregulated *C. briggsae* alleles.

These findings suggested that in terms of sex-related gene expression, the *C. nigoni* haplotype exhibited an elevated expression level in the F1 hybrids compared with the *C. briggsae* haplotype.

### NB Showed a Higher Expression Level of TEs than did BN

**Fig. 6.**
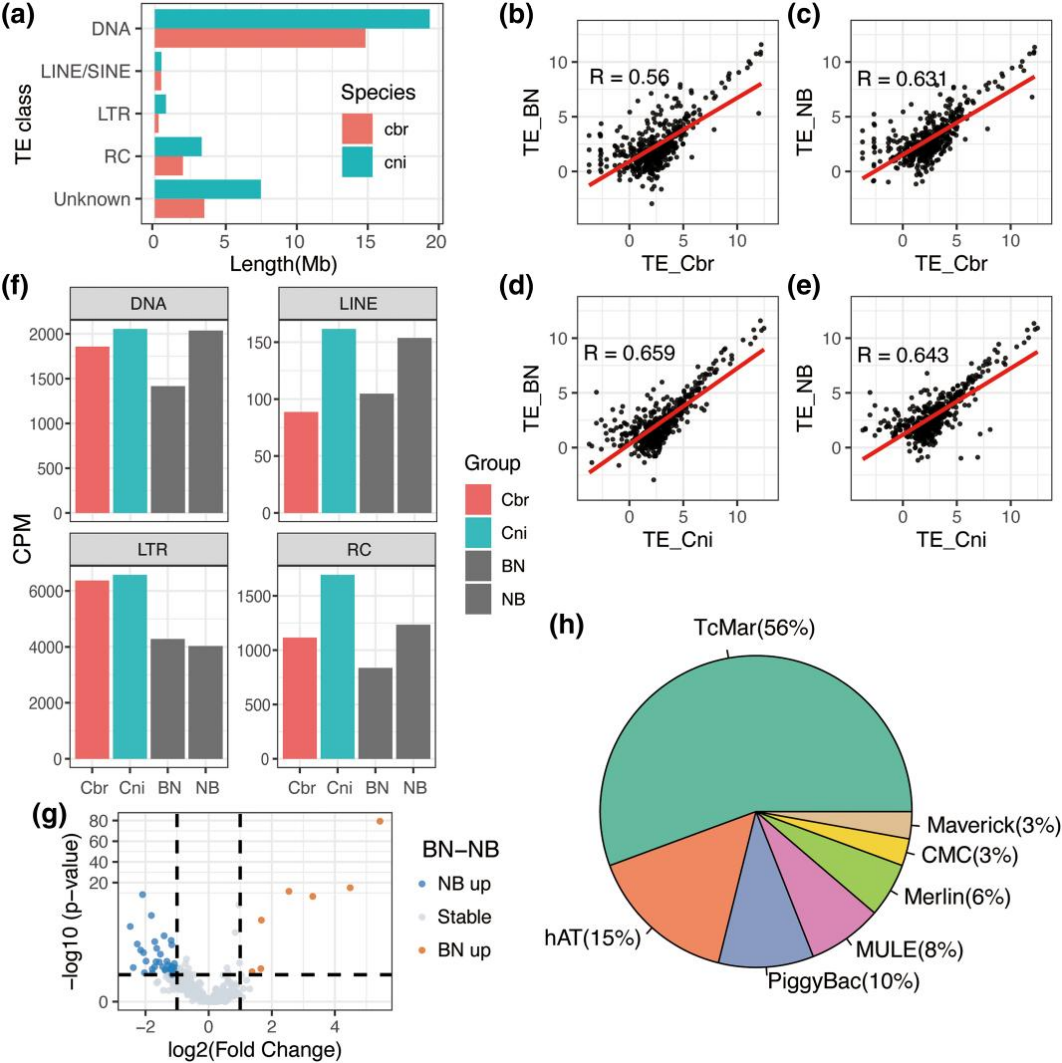
Comparison of TE expression between the two F1 hybrids and parental species. a) Bar plot comparing the overall lengths of four main TE classes in *C. briggsae* and *C. nigoni* at the genome level. b to e) Spearman correlation of all the TEs in the two F1 hybrids and wild-type young adult female worms: Spearman correlation between wild-type *C. briggsae* and its haplotype in BN (b) and NB (c); Spearman correlation between wild-type *C. nigoni* and its haplotype in BN (d) and NB (e). f) Bar plots comparing the overall expression of TEs in females at the TE family level between *C. briggsae*, *C. nigoni*, BN, and NB in different TE classes, namely DNA, LINE/SINE, LTR, and RC. g) Volcano plot of the differential TE families between BN and NB. h) Pie chart in which DNA TEs are further divided into eight subclasses. LINE, long interspersed nucleotide element; SINE, short interspersed nuclear element; LTR, long terminal repeat; RC, rolling circle.

The derepression of TEs in F1 offspring is often linked to hybrid dysfunction (Kelleher et al. 2012; Guerreiro 2014). We therefore performed an analysis of the TE transcripts in the two directions of F1 hybrids between *C. nigoni* and *C. briggsae*. The TE classes from the genomes of *C. nigoni* and *C. briggsae* are primarily composed of DNA and rolling circle (RC) elements (Fig. 6a). Through mapping RNA-seq reads to the TEs’ consensus sequences, we obtained 874 families in *C. briggsae* and 1,019 families in *C. nigoni* (supplementary table S9, Supplementary Material online). After filtering out the low-expression families, 472 families were used for further analysis, of which 282 belonged to *C. nigoni* and 190 belonged to *C. briggsae*. The expression correlation of TE families between the two parent species was low (supplementary fig. S10a, Supplementary Material online), whereas the correlation between the two hybrids was highly consistent (supplementary fig. S10b, Supplementary Material online). The TE expression in both BN and NB showed a stronger correlation with *C. nigoni* than with *C. briggsae* (Fig. 6b to e). Additionally, the TE expression in NB exhibited a stronger Spearman correlation with *C. briggsae* compared with BN, potentially reflecting maternal effects.

The expression of TEs in the hybrids did not surpass the levels observed in either parental species (Fig. 6f). NB exhibited a higher TE expression level than *C. briggsae* in terms of DNA, long interspersed nucleotide elements (LINEs) and RC. In addition, we found that a long terminal repeat (LTR) family discovered in *C. briggsae*, rnd-1_family-457#LTR/Gypsy, was highly expressed in both species and in the F1 hybrids, and contributed to the majority of the LTR TE reads.

TE expression was higher in NB than BN, as indicated by the TE classes of DNA, LINE, and RC elements (Fig. 6f). According to the differential expression analysis between BN and NB in terms of TEs (*P* < 0.05, |log_2_ fold change| > 1; Fig. 6g), there were 50 differentially expressed families between the two hybrids, with 42 and 8 families showing a higher expression level in NB and BN, respectively. Among the eight more highly expressed families in BN, six exhibited a significant fold change.

In previous studies, MULE DNA, which is a TE in *C. briggsae*, was reported to confer a hybridization advantage in the HK104 strain by incorporating a toxin-antidote (TA) element, prompting our further analysis of DNA TEs (Widen et al. 2023). The 177 expressed DNA TEs could be grouped into seven transposon types, predominantly TcMar (56%), followed by hAT (15%), PiggyBac (10%), and MULE (8%; Fig. 6h). Among the 13 categorized *C. briggsae*–specific TE families that were not expressed in *C. nigoni* (count per million [CPM] < 1), 7 were from TcMar and 3 from MULE. Notably, the rnd-1_family-292.cb5.DNA/TcMar was uniquely expressed in NB, unlike the five TE families exclusive to *C. nigoni.* Overall, no abnormal activation of TEs was observed in the two F1 hybrids.

## Discussion

The *C. briggsae–C. nigoni* pair has been established as a model for understanding the genetic basis of speciation. A previous study found several phenotypic asymmetries in these hybrids, including a significant difference in viability between the two hybrid directions, as well as directional survival of F2 offspring, both indicating a bias toward hybrids that carry more genetic materials from one parental species, namely *C. nigoni* (Woodruff et al. 2010). This phenotypic asymmetry provided insights into the underlying genetic mechanisms of the reproductive barriers between the two species and the evolutionary processes driving their divergence. Previous research on transcriptome F1 hybrids between *C. briggsae* and *C. nigoni* has focused on the regulatory dysfunction and sex-biased gene expression between F1 males and F1 females within the same crossing direction (Sanchez-Ramirez et al. 2021). However, studies have not yet addressed the mechanisms behind the distinct phenotypes of F1 female *C. briggsae–C. nigoni* hybrids from the two crossing directions. Examining the patterns of gene expression and phenotypic variation in hybrid offspring from reciprocal crossings is expected to allow a better understanding of the molecular mechanism underlying hybrid dysfunction and speciation.

Reciprocal crossing has been explored in various systems. Pioneering work by Wittkopp et al. observed that in *Drosophila melanogaster* × *Drosophila simulans* female hybrids (Wittkopp et al. 2004), the cross direction had a negligible effect on ASE for most genes, indicating that F1 transcriptomes are largely equivalent when hybrid viability is high, consistent with the inheritance and regulatory profiles in our study. Likewise, research on *Nasonia vitripennis* and *Nasonia giraulti*, along with their reciprocal viable F1 female hybrids (Wang et al. 2016), revealed that most genes showed no parent-of-origin bias, but some genes associated with methylation were predominantly driven by *cis*-regulatory elements. In organisms with parent-of-origin epigenetic regulation, the direction of the cross can be critical. For example, in a *Drosophila pseudoobscura* species pair exhibiting unidirectional hybrid sterility, sterile male hybrids had more misregulated and more over- or underexpressed genes relative to the parental species than the fertile male hybrids (Gomes and Civetta 2015). Here, we investigated ASE in hybrid F1 females derived from reciprocal crosses between *C. briggsae* and *C. nigoni*, which are both fertile but showed different viability and fertility, aiming to elucidate the molecular mechanisms underlying the phenotypic asymmetries observed in the hybrid progeny.

The first and most significant observation is that while both directional F1 female hybrids are fertile, the viability of BN is considerably higher than that of NB. This is consistent with the weak inbreeder/strong outbreeder (WISO) hypothesis (Brandvain and Haig 2005), according to which, in crosses between species with different mating systems, outcrossing parent species will “overpower” selfing parents such that their genomes have a higher “effective ploidy” in hybrid offspring (Xie et al. 2022). Initially, we sought to understand this phenomenon by examining the inheritance expression. Except for no change (27.5% to 37.5%), most transcripts that are differentially expressed fall into one of the dominant categories (41% to 42%), followed by transgressive (overdominant or underdominant; 15% to 23%) and additive modes of inheritance (6% to 7%). In contrast to the inheritance study on BN females (Sanchez-Ramirez et al. 2021), we observed a higher proportion of dominant categories relative to transgressive genes. This discrepancy may be attributed to differences in experimental design, RNA-seq analysis workflows, and applied statistical thresholds. Furthermore, a similarly high proportion of one of the dominant categories has also been described in hybrids of *Drosophila* species (McManus et al. 2010; Díaz et al. 2023). Since transgressive phenotypes are often assumed to result from transgressive gene expression (Rieseberg et al. 2003; Renaut et al. 2009), the relatively lower incidence of transgressive genes in our study could reflect the fact that both BN and NB female hybrids remain viable and fertile. Nevertheless, we did identify a higher proportion of transgressive genes in NB than in BN, implying that overdominant or underdominant regulatory mechanisms were still at play in this hybrid combination.

We found that the gene expression patterns of both BN and NB showed a stronger correlation with *C. nigoni* than with *C. briggsae*, and *C. nigoni*–dominant orthologs also outnumbered those of *C. briggsae*. We surmised that this may be due to the stronger influence of the *C. nigoni* haplotype in the F1 hybrids than that of *C. briggsae*. Building on this, we also found that, influenced by the maternal effect, the expression correlation of NB with *C. briggsae* was stronger than that of BN, potentially leading to more pronounced internal conflicts between alleles from the two parental species, reflecting in more transgressive orthologs in NB. This is one of the underlying reasons for the reduced viability observed in NB. We also found genes more highly expressed in F1 hybrids than in either parental species (overdominant and additive) were more likely to accumulate nonsynonymous mutations leading to amino acid changes, whereas genes expressed at lower levels (underdominant) were less likely to exhibit such mutations. Previous observations showed that the rate of gene (or protein) sequence evolution was negatively correlated with the gene expression level (Liao and Zhang 2006; Chu and Wei 2019). However, these studies did not investigate the offspring of two species in relation to their parent species. At present, although the specific functions of additive genes remain unclear, overdominant expression did not impact fitness as negatively as regulatory divergence that leads to underdominance in hybrids (Sanchez-Ramirez et al. 2021). We suspected that a high rate of nonsynonymous substitutions might indicate that these mutations exert a beneficial effect on BN and NB hybrids, leading to their retention.

In addition, due to the essential role played by maternal effects in female hybrids, mitochondria—which bear maternal inheritance—are an indispensable consideration when studying genetic asymmetry. Nuclear–cytoplasmic incompatibility also is a crucial part of the Dobzhansky–Muller model (Barr and Fishman 2010). The mitochondria of the two species drive the evolution of mitochondrial-related, nuclear-encoded genes, which may subsequently affect the transcriptome of hybrids resulting from interspecies crosses. Here, we posited that nuclear–cytoplasmic incompatibility exists in the two female hybrids, with it being more pronounced in the NB direction. We showed the *cis*-regulated genes in both mitochondria-encoded and nucleus-encoded mitochondrial genes are more dominant, compared with the overall nucleus-encoded genes. In terms of inheritance profile, the proportion of additive mitochondrial orthologs is higher compared with that of overall orthologs. In the comparison of nuclear genes between BN and NB, we observed that highly expressed in NB compared with BN enriched in pathways related to mitochondrial functions. The upregulation of specific mitochondrial-related genes in NB possibly served as a compensatory mechanism or functional dysfunction, which could be related to the complementary gene interactions described in the Dobzhansky–Muller model. We propose that the suppression of haplotype fusion in F1 hybrids may be driven by transcriptome regulation, which appears to mitigate the maternal effects of mitochondria. This suggests a complex interplay at the transcriptional level where nuclear genes may compensate for mitochondrial influences. Furthermore, the more pronounced adverse effects of nuclear–cytoplasmic incompatibility observed in NB than in BN likely arise from mismatched interactions between mitochondrial gene expression, which is predominantly controlled by *C. briggsae*, and nuclear gene expression, which is largely influenced by *C. nigoni*. This hypothesis is supported by the observed correlations and inheritance patterns that distinguish the effects of nuclear–cytoplasmic incompatibility from purely maternal mitochondrial effects.

Despite a relatively lower proportion of transgressive genes in hybrid females compared with the previous work (Sanchez-Ramirez et al. 2021), we have also observed that underdominant genes have the highest proportion on the X chromosome, especially in NB, which were explained as suppression to male-biased expression. Upon discovering that overdominant genes typically exhibit female-biased expression, we also consider that overdominant expression does not negatively impact fitness to the same extent (Sanchez-Ramirez et al. 2021). Intriguingly, our analysis further revealed that the BN exhibited a stronger female bias in overdominant genes than NB (Fig. 2i), suggesting that overdominance may contribute more significantly to the enhanced fitness of BN hybrids. This hypothesis was supported by the fact that BN demonstrated greater viability than NB (Woodruff et al. 2010). Furthermore, we also observed an enrichment of male-biased terms in the underdominant genes in BN and NB hybrids (Fig. 2i), which possibly drove the hybrids toward a strictly female phenotype rather than a hermaphroditic one (Sanchez-Ramirez et al. 2021). This finding was consistent with the cryptic masculinization observed in the overtly female soma and oogenic germline of *C. briggsae* hermaphrodites (Thomas et al. 2012). Moreover, genes only underdominant in BN showed significant enrichment in hermaphrodites may point out a stronger cryptic masculinized compared with NB. These findings suggested that differences in how sex-related genes are affected by the direction of crossing may also be a potential factor contributing to the observed disparities in viability between BN and NB hybrids.

Another phenotypic asymmetry discovered by Woodruff and his team in the hybridization of *C. nigoni* and *C. briggsae* was that only when F1 females from both directions crossed with male *C. nigoni* produced viable F2 progeny (Woodruff et al. 2010). In contrast, crosses with *C. briggsae* male did not yield viable progeny. The raises the question of why NB hybrids, despite being influenced by the maternal effect, failed to produce viable offspring with its mother species. Our findings suggest that the *C. nigoni* haplotype exhibits a more dominant expression profile for sex-related genes in F1 hybrids compared with *C. briggsae*. Specifically, among the differentially expressed genes (DEGs) shared by BN and NB hybrids, only orthologs with elevated expression in *C. nigoni* alleles were significantly enriched in oogenic and female-biased gene sets. Additionally, more orthologs that were more highly expressed in *C. nigoni* alleles belonged to the X chromosome, compared with *C. briggsae*. The *C. nigoni* haplotype dominance of sex-related genes may be related to the fact that BN and NB are female rather than hermaphrodites, suggesting a potential link between sexual mode divergence and gene expression dominance. This aligns with the notion that female-typical genes may be less canalized in species with hermaphroditic gonads, allowing *C. nigoni* alleles to be more robustly expressed in hybrids.

In terms of regulatory profiles, *trans*-regulated genes showed significant enrichment in male-biased and spermatogenesis categories, which was also found in previous results (Sanchez-Ramirez et al. 2021). This finding suggests that the *trans* effect may initially impact genes with male characteristics or those with dual-gender functions, which may also be key in promoting convergence in hybridization between the two species. In contrast, the *cis*-regulated gene sets were more biased toward females. Furthermore, orthologs affected by *trans-*regulatory effects exhibit higher *K*_a_/*K*_s_ values, indicating that these genes may be subject to stronger positive selection or relaxed purifying selection. Regarding the relationship between regulatory types and inheritance profiles, we found that additive orthologs exhibited a higher proportion of *trans*-regulated genes than other inheritance categories. This discovery was not consistent with the finding in previous work that additive genes were highly related to *cis-*regulatory divergence (Lemos et al. 2008; McManus et al. 2010). However, this link was not necessarily universal (Coolon et al. 2014). We speculated that multiple *trans*-acting factors might collectively influence gene expression, ultimately resulting in additive outcomes, which is also consistent with a previous finding that *trans* effects accounted for a greater proportion of the regulatory divergence at sites with additive compared with nonadditive inheritance patterns (Bell et al. 2013).

Abnormal activation of TEs in hybrids of two species is also considered a crucial factor for HI in some species (Metcalfe et al. 2007; Shpiz et al. 2014; Erwin et al. 2015). For *C. nigoni* and *C. briggsae*, the dysregulation of repetitive elements has been postulated as a potential reason for the difference in genome size, which differs by >20 Mb. However, a previous study showed that there are no differences in the global expression levels of TEs between hybrid sterile males from two introgression lines and *C. nigoni*. In our own research on TEs, we found that, consistent with the previous finding, there is no increased expression of TEs in F1 hybrids compared with the two parental species. Additionally, we observed a higher expression level of DNA transposons, LINEs, and RCs in backcross hybrids of F1 with *C. nigoni* (NB) compared with backcross hybrids of F1 with *C. briggsae* (BN). Therefore, the F1 hybrids may not be significantly affected by TE expression.

## Conclusion

We analyzed the transcriptomes of parents and F1 female hybrids between *C. briggsae* and *C. nigoni* from reciprocal crosses. Comparing the expression in the hybrids with that in the parental species, we found that the *C. nigoni* genes maintained their expression pattern better than the *C. briggsae* genes in the two F1 female hybrids, which was consistent with the WISO hypothesis. This difference also occurred in the expression of sex-biased genes, which more closely resembled those of *C. nigoni* than those of *C. briggsae.* Additionally, the results related to mitochondrial genes suggested the presence of nuclear–cytoplasmic incompatibility in the hybrids, primarily manifesting as *cis*-dominated expression of mitochondrial genes. Notably, the NB progeny exhibited a greater degree of this incompatibility, potentially contributing to lower viability in NB compared with BN. Although there was no abnormal activation of TEs at the TE level, TE expression in NB was higher than in BN.

## Methods

### Samples, RNA Isolation, and Illumina Sequencing

All worm strains were maintained on NGM plates at 25 °C with preseeded OP50 *Escherichia coli*. Three hundred young adult females were collected for mRNA extraction for each sample of *C. briggsae* (AF16), *C. nigoni* (JU1421), and the F1 female hybrids BN and NB. “BN” indicates the F1 female hybrid from the crossing of *C. briggsae* males and virgin *C. nigoni* females; “NB” indicates the F1 female hybrid from the crossing of *C. nigoni* males and sperm-depleted *C. briggsae* hermaphrodites. Total RNAs were extracted using TRIzol Reagent (Invitrogen, MA, USA), following the manufacturer's instructions. mRNA purification and fragmentation, cDNA synthesis, end repair, adapter ligation, and DNA fragment enrichment were performed using Illumina's TruSeq-stranded mRNA library preparation kit according to the kit's manual. Each library was barcoded and sequenced to obtain paired-end (2 × 150 bp) reads using an Illumina MiSeq. The *C. nigoni* samples were sequenced in triplicate, while the other samples were sequenced in duplicate (supplementary table S1, Supplementary Material online).

### Analysis of RNA-Seq Reads for mRNA Expression

The adaptor sequences of raw mRNA-seq reads were first trimmed using trimmomatic v0.39 (the used adaptor reference is TruSeq3-PE.fa; Bolger et al. 2014), and the low-quality reads (Phred quality score <20) were filtered out. The processed reads were assessed using FastQC v0.12.1 (https://github.com/s-andrews/FastQC) for quality control.

To ensure a fair mapping of mRNA-seq data to both genomes, we combined the two genome assemblies, *C. briggsae* (cb5) and *C. nigoni* (cn3) as a new reference genome for hybrid RNA-seq reads mapping. The respective gene annotation files in General Feature Format (GFF3) were also combined together for the mapping. The final mapping index for tophat2 consisted of a FASTA file, containing the genomes of both species and a combined GFF file that includes annotations for both species. The combined genomes and gff3 files can be found in the folder “./reference/transcriptome” in the supplementary link.

We mapped the trimmed and quality-controlled mRNA-seq reads to the prepared genome using tophat2 v2.1.1 (Kim et al. 2013) and bowtie2 v2.2.5 (Langmead et al. 2009) with the default parameters. Before mapping, specific annotation indexes for the combined genome FASTA file were built by bowtie2 (https://bowtie-bio.sourceforge.net/tutorial.shtml), and combined GFF indexes were built by tophat (https://ccb.jhu.edu/software/tophat/tutorial.shtml). The mRNA-seq reads were mapped through the official recommended pipeline with default parameters (https://ccb.jhu.edu/software/tophat/manual.shtml). After obtaining the mapped BAM files, we used HTSeq-count v2.0.2 (Anders et al. 2015) with the default parameter to calculate raw counts for each gene, with “intersection-nonempty” mode and “-type” was exon. Multiple alignments and alignments whose mapping quality is equal to 0 were filtered after counting the HTSeq-count. All raw count tables were in the folder “./raw_data/transcriptome/” in the supplementary link. We used a custom Python script to check whether each read's mapped genome alignment (mapping quality > 0) originated from the same and expected species (see file./codes/Figure 1_ambiguity.py in the supplementary link, related tables were in folder “ambiguity” in the supplementary link).

### Analysis of RNA-Seq Reads for Mitochondria-Encoded Genes Expression

We used BWA-MEM (Li 2013) to map the RNA-seq data to the mitochondrial genomes of two species (Li et al. 2018; supplementary table S6, Supplementary Material online). Raw counts were obtained by HTSeq-count v2.0.2 with “intersection-nonempty” mode and were further normalized to reads per kilobase of exon model per million mapped reads. The count tables were in the folder “./raw_data/mitochondria/” in the supplementary link.

### Analysis of RNA-Seq Reads for TE Expression

TE annotations were obtained from Repeat Modeler v2.0.3 (Flynn et al. 2020) and Repeat Masker (Tarailo-Graovac and Chen 2009). The consensus FASTA file of *C. briggsae* and *C. nigoni* produced by Repeat Masker was combined as the TE consensus reference (supplied in folder “./reference/TE” in the supplementary link). In total, we detected 579 *C. briggsae* and 518 *C. nigoni* TE families, including DNA, LINE, LTR, RC, and short interspersed nuclear element (SINE). Raw data were mapped to the combined TE consensus reference by BWA-MEM (supplementary table S9, Supplementary Material online), and mapped bams were counted by HTseq v2.0.2 with “intersection-nonempty” mode. The related middle files were in folder “./raw_data/TE” in the supplementary link.

### Ortholog Identification

We used mutual best BLASTP hits to obtain one-to-one orthologous genes for transcriptome analysis (*e*-value <1*e*^4^, supplementary table S2, Supplementary Material online; Altschul et al. 1990; Ren et al. 2018). To eliminate the influence of isoforms, we retained only the longest gene at the same position on the chromosome and kept its longest protein. The longest proteins for each gene were used as the input for BLASTP. The middle files were in folder “ortholog” in the supplementary link.

After we obtained one-to-one orthologs, we used paraAT (Zhang et al. 2012) and KaKs_calculator 2.0 (Wang et al. 2010) with clustalw2 mapping tools (https://github.com/sochix/parallel-clustalw2) to obtain the *K*_a_/*K*_s_ values (supplementary table S3, Supplementary Material online). Orthologs with *K*_a_/*K*_s_ values >1 were listed with the length of the transcripts (supplementary table S4, Supplementary Material online). The higher this value, the higher the possibility of adaptive evolution.

To identify one-to-*n* mitochondria-related orthologs, all protein-coding genes from *C. briggsae* and *C. nigoni* were blasted against the nuclear-encoded mitochondrial genes from Human MitoCarta3.0 (Rath et al. 2021) using BLASTP (supplementary table S5, Supplementary Material online) with a cutoff of *e*-value <1*e*^−4^. One human nuclear-encoded mitochondrial gene term could have multiple homologs. The gene expression for homologs in each human term was summed for downstream analysis. The used code was in the supplementary link (“./codes/Figure 4_mitochondria_new.R”).

### Statistical Analysis

We used the R (v4.3.1) package edgeR v3.38.4 (Robinson et al. 2010) to quantify differential expression. Before statistically assessing differential expression, we rearranged and summed the allele-specific counts for F1 hybrids to obtain CPM values according to one-to-one orthologs. For parental species, counts mismatched with haplotypes from other species were deleted, resulting in the formation of distinct groups: Cni, Cbr, BN_cbr, BN_cni, NB_cbr, and NB_cni. Cni contained three replicates, while the other groups each contained two replicates. We filtered out genes unexpressed in at least 4 samples, requiring CPM values higher than 2 in 13 library-size scaled counts. This filtering criterion was consistently applied in all subsequent differential expression analyses. Regarding the normalization of data for mitochondria-coded genes and TEs, all raw counts were compiled into a single normalization background. Additionally, for mitochondria-related genes, normalization was performed using only the sum of 4,547 genes identified as related to mitochondria.

Fisher's exact test was also used to determine the significance of the numerical differences between two selected categories, such as chromosomes and inheritance patterns, with |log_2_ odds ratio| > 0.4 and *P* < 0.01 considered as significant.

### Modes of Expression Inheritance in F1 Hybrids

To investigate the patterns of expression in F1 hybrids relative to the parental species, we classified genes into those having additive (intermediate), dominant (matching either of the species), overdominant (higher than both parents), and underdominant (lower than both parents) profiles following the logic of previous studies (McManus et al. 2010; Sanchez-Ramirez et al. 2021). Here, we first used edgeR v3.38.4 (Robinson et al. 2010) to compare the expression of each ortholog between each F1 hybrid and each parent species. We used the cutoff values of FDR = 0.05 and |log_2_ fold change| = 1 to classify the 10,381 expressed genes. If the *P*-values for hybrids and both parent species were >0.05 and the absolute log fold changes for both conditions were <1, the gene is classified as “no change.” If one ortholog showed no significant difference between hybrids and one of the parent species (FDR > 0.05 and |log_2_ fold change| < 1), the ortholog was labeled as “dominant.” If one ortholog showed lower expression in hybrids than both parent species, the ortholog was considered “underdominant” (FDR < 0.05 and log_2_ fold change < 0). If one ortholog showed higher expression in hybrids than both parent species, the ortholog was termed “overdominant” (FDR < 0.05 and log_2_ fold change > 0). Otherwise, if none of the above conditions were met, the ortholog was classified as “additive.” The used code was in the supplementary link (./codes/Figure 2_inheritance_new.R).

### *Cis*- and *trans*-Regulated Divergence

We used ASE in the two F1 hybrids separately to obtain the *cis*- and *trans*-regulated profile between the two species. We classified our data according to the following traditional definition of *cis* and *trans* effect (Wittkopp et al. 2004; Mack and Nachman 2017). When there were no significant DEGs (FDR > 0.05) in either the parental (P) data sets or two F1 hybrids (H) data sets, these genes were considered conserved. Species-specific mRNA abundance ratios between the P and H were further compared with classified *cis* and *trans* alleles. Genes were classified as predominantly influenced by *cis*-regulatory effects when they showed significant differential expression either in P or in H and its mRNA abundance ratio difference between the P and H showed minor divergence (customized as absolute difference < 1.8). The remaining genes were considered influenced by *trans*-regulatory effects. The proportion of regulatory and inheritance was calculated by the number of shared regulatory and inheritance categories divided by the overall number of the corresponding inheritance category. The used code was in the supplementary link (./codes/Figure 3_regulatory.R).

### Imprinted Gene Detection and Validation

The cutoff used to define paternal/maternal imprinted genes was an allele from the paternal/maternal part consisting of ≥75% of the ortholog pair in both directions of F1 hybrids.

Detected imprinted genes were validated with the previously annotated genes on the imprinted gene database at https://www.geneimprint.com/. The amino acid sequences of the detected imprinted genes were compared with the genes annotated by Blastp (*e*-value <1*e*^4^). Only genes that could be mapped in both directions were confirmed as validated imprinted genes.

### Enrichment Analysis of Misexpressed Orthologs

The enrichment analysis used in this study included sex-related gene sets, GO, and the KEGG. We used the R package ClusterProfiler v4.8.3 (Yu et al. 2012) to analyze different gene sets for GO and KEGG enrichment. We first obtained the annotation of *C. briggsae* and then established the corresponding information for *C. nigoni* by considering one-to-one orthologs. GO annotations were obtained from Wormbase (c_briggsae.PRJNA10731.WS279.go_annotations.gaf.gz). Finally, we obtained KEGG data on *C. briggsae* from the KEGG official (KEGG BRITE: KEGG Orthology—*C. briggsae* [nematode]). The *P*-value and FDR were calculated for each category, and the categories with FDR < 0.05 were reported.

To infer genes involved with gonad functions, we downloaded the annotation of *C. elegans* spermatogenesis and oogenesis genes from previous research (Ortiz et al. 2014). The gonad categories were obtained by comparing the expression of *fem-3* spermatogenic to *fog-2* oogenic gonads, including spermatogenesis (logFC > 1, FDR < 0.01), oogenic (logFC < −1, FDR < 0.01), and gender neutral. The annotations were transferred to *C. briggsae* genes by using the one-to-one orthologs between *C. elegans* and *C. briggsae*. We obtained the ortholog annotation between *C. elegans* and *C. briggsae* from Wormbase (https://downloads.wormbase.org/species/c_elegans/PRJNA13758/annotation/orthologs/). Regarding sex-specific categories, we used the RNA-seq gene expression matrix from two *C. briggsae* datasets involving the comparison between males and hermaphrodites to obtain the gene annotation for sex-specific categories, respectively (Albritton et al. 2014; Sanchez-Ramirez et al. 2021). The gene annotation from Albritton et al. (2014) contained seven terms, namely female specific (FemSpec, FPKM >1 and only expressed in female), high female (HighFem, female compared with male log_2_ FC > 3), low female (LowFem, female compared with male 1 < log_2_ FC <3), no sex biased (NoBias, *q*-value >0.05 and |log_2_ FC| < 1), low male (LowMale, male compared with female 1 < log_2_ FC <3), high male (HighMale, male compared with female log_2_ FC > 3), and male specific (MaleSpec, FPKM >1 and only expressed in male), which was defined based on the differential expression between *C. briggsae* males and pseudo-females (AF16-derived she-1(v47) mutant strain). The gene annotation from (Sanchez-Ramirez et al. 2021) contained female-biased, sex-neutral, hermaphrodite, and male-biased terms, which also compared the *C. briggsae* hermaphrodites to pseudo-females following the differential expression method in Thomas et al. (2012). The two sex-specific gene annotations were used independently for our enrichment analysis. The *P*-value and FDR were calculated for each sex-specific category using the enricher function (R package ClusterProfiler v4.8.3; Yu et al. 2012), and the categories with FDR < 0.05 were reported.

## Supplementary Material

evaf098_Supplementary_Data

## Data Availability

All sequencing data generated in this article have been deposited in the NCBI database at the following URL: https://www.ncbi.nlm.nih.gov/bioproject/PRJNA1125123. All middle files and used codes were provided in the supplementary link on FigShare (https://doi.org/10.6084/m9.figshare.28294436.).
