# Supplementary material for: RNA-sequencing Analysis of Hybrid Females Reveals a Dominance of Expression of Alleles From Outcrossing Species Over Those From Selfing Species
Source: Genome Biol Evol. 2025 Jun 6;17(6):evaf098. doi: 10.1093/gbe/evaf098 (PMC12142003; doi:10.1093/gbe/evaf098)
Supplement: evaf098_Supplementary_Data [file evaf098_supplementary_data.zip › Figure Suppli.pdf]

Supplementary Figures

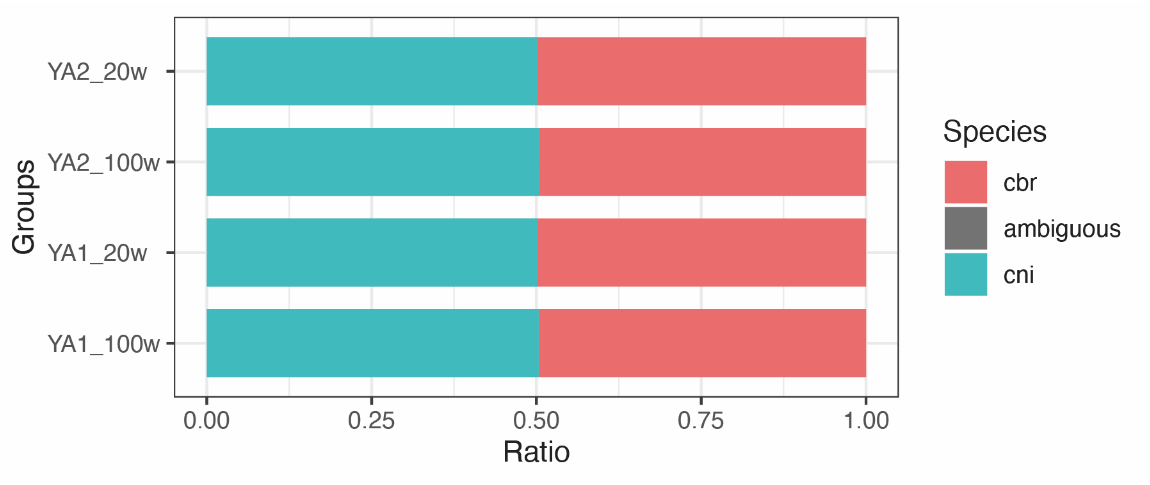

Figure S1: A simulation diagram of mapping ambiguity after mixing an equal number of reads from the parent species data of two replicates into the same FASTQ reads file, simulating the cases where 200,000 reads and 1,000,000 reads are selected for each species. The proportion was around 50:50.

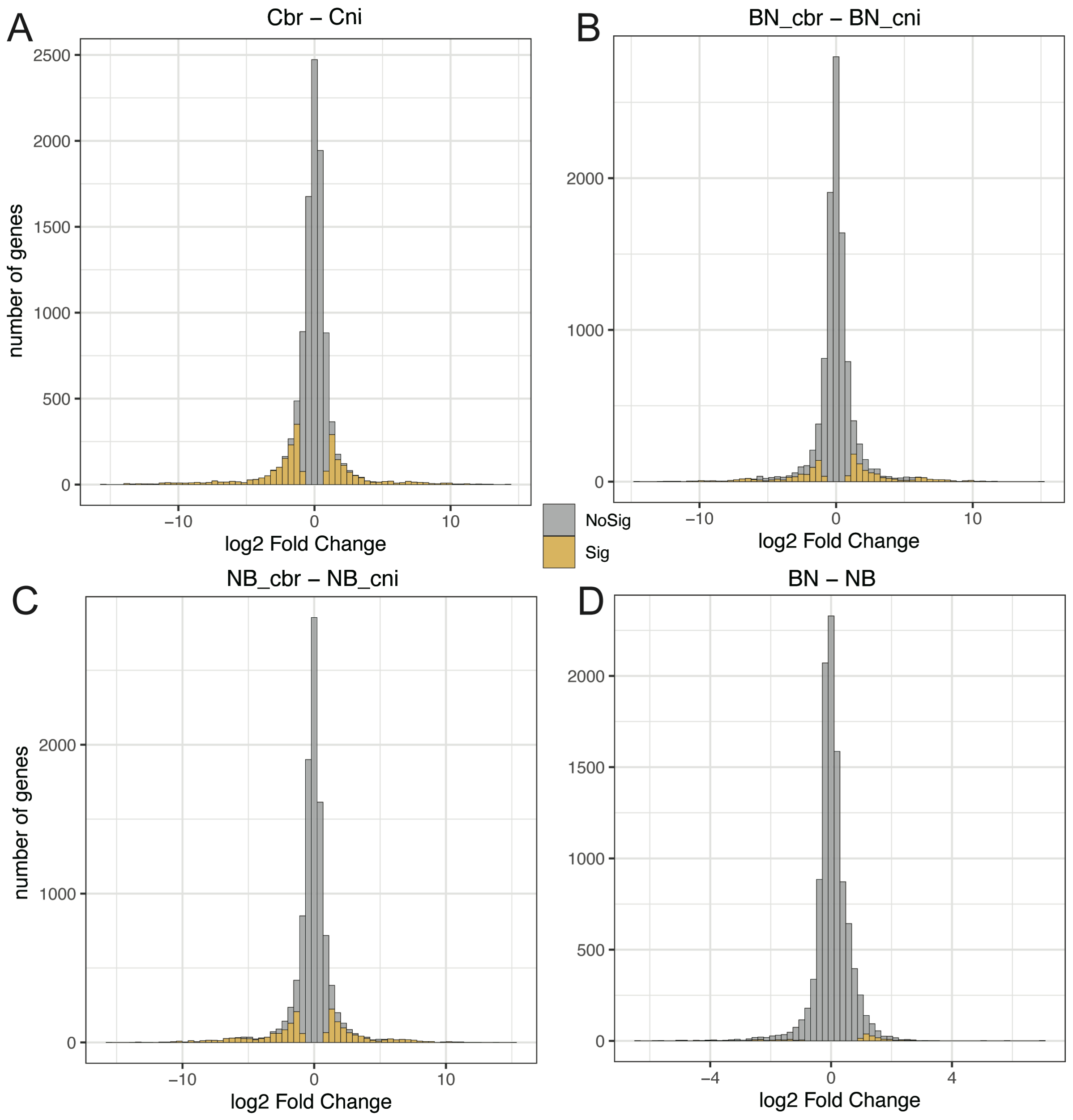

Figure S2: Density plot shows differential distributions of gene expression between *C. briggsae* and *C. nigoni* (A), BN_cbr and BN_cni (B), NB_cbr and NB_cni (C), BN and NB (D). Cni, *C. nigoni*; cbri, *C. briggsae*; BN, the F1 female hybrid from the crossing of *C. briggsae* male and *C. nigoni* female; NB, the F1 female hybrid from the crossing of *C. nigoni* male and *C. briggsae* hermaphrodite; BN_cni, the *C. nigoni* haplotype inside the young adult BN; BN_cbr, the *C. briggsae* haplotype inside BN; NB_cni, the *C. nigoni* haplotype inside the young adult NB; NB_cbr, the *C. briggsae* haplotype inside NB.

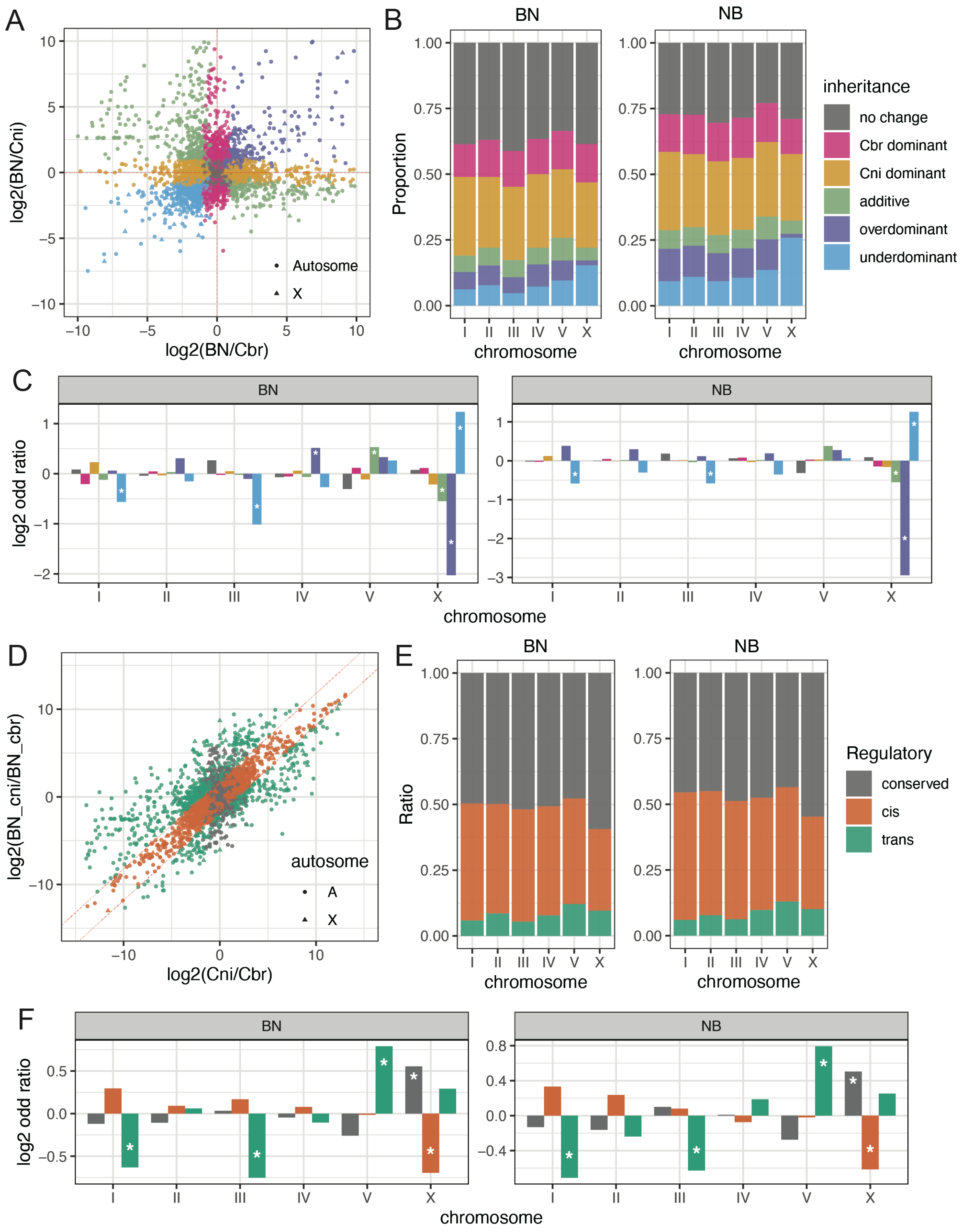

Figure S3: Chromosome distribution of expression inheritance and regulatory profile. (A-C) Chromosome distribution of expression inheritance modes between two haplotypes in F1 hybrids. (A) The dotplot showing the method used to define the expression inheritance categories, including additive, Cbr dominant, Cni dominant, no change, overdominant and

underdominant genes, between BN hybrids and two parental species. (B) The distribution of genes with different inheritance profiles among different chromosomes of two F1 hybrids. (C) Enrichment of gene sets with different inheritance profiles in different chromosome with Fisher's exact test, log2 odds ratio, i.e. the log2 transform of observed/expected, P value < 0.01 and |log2 odds ratio| > 0.4. (D-F) Chromosome distribution of expression inheritance modes between two haplotypes in F1 hybrids. (D) The dot plot showing the method used to define the *cis*-, *trans*-, or conserved genes inside BN. (E) The distribution of *cis*, *trans* and conserved genes among different chromosomes of two F1 hybrids. (F) Two F1 hybrids' enrichment of genes in six chromosomes with different regulatory profiles by Fisher's exact test, log2 odds ratio, i.e. the log2 transform of observed/expected, P value < 0.01 and |log2 odds ratio| > 0.4.

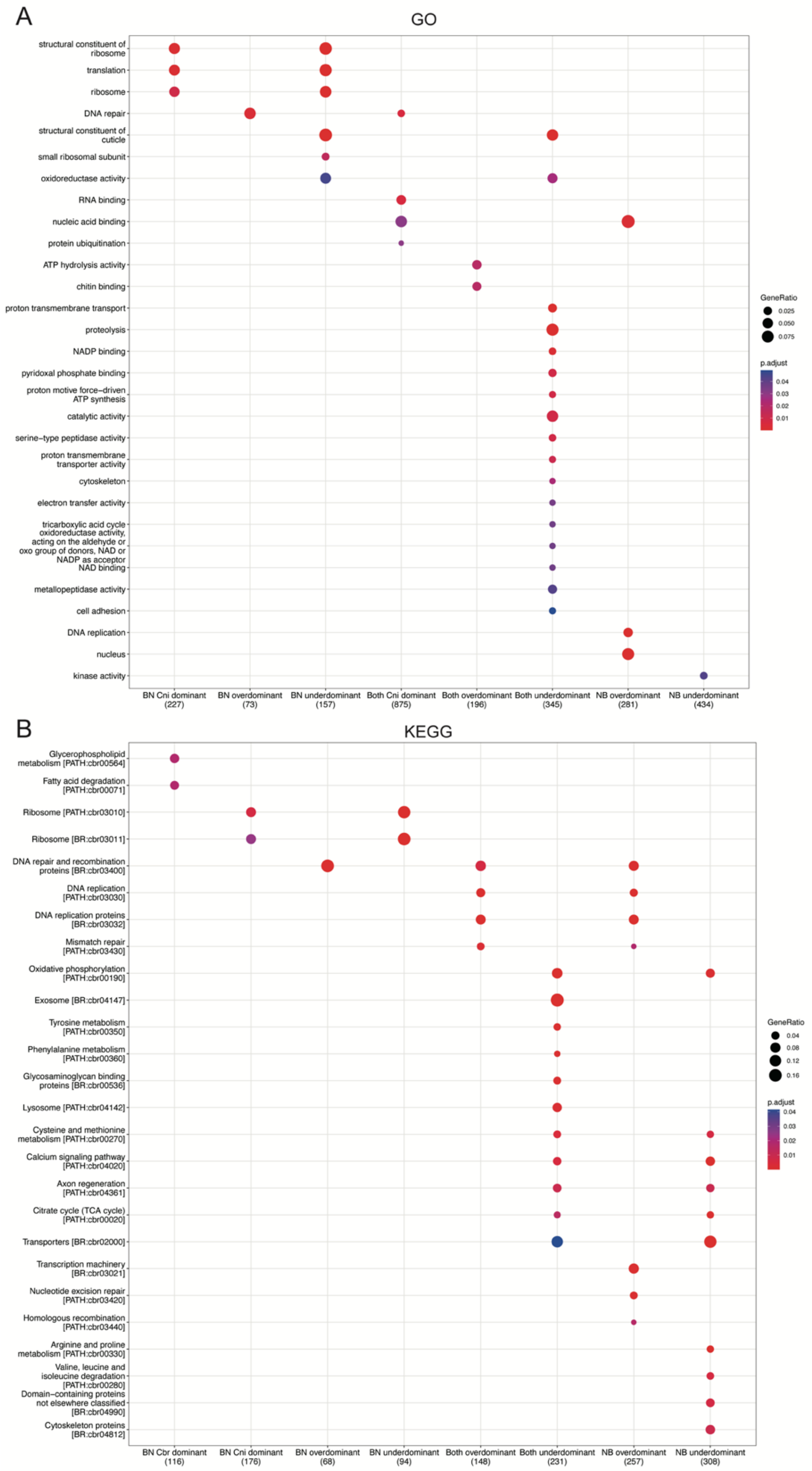

Figure S4: Gene ontology (GO) (A) and Kyoto Encyclopedia of Genes and Genomes (KEGG) (B) pathway enrichment according to inheritance profiles clustered by two F1 hybrids.

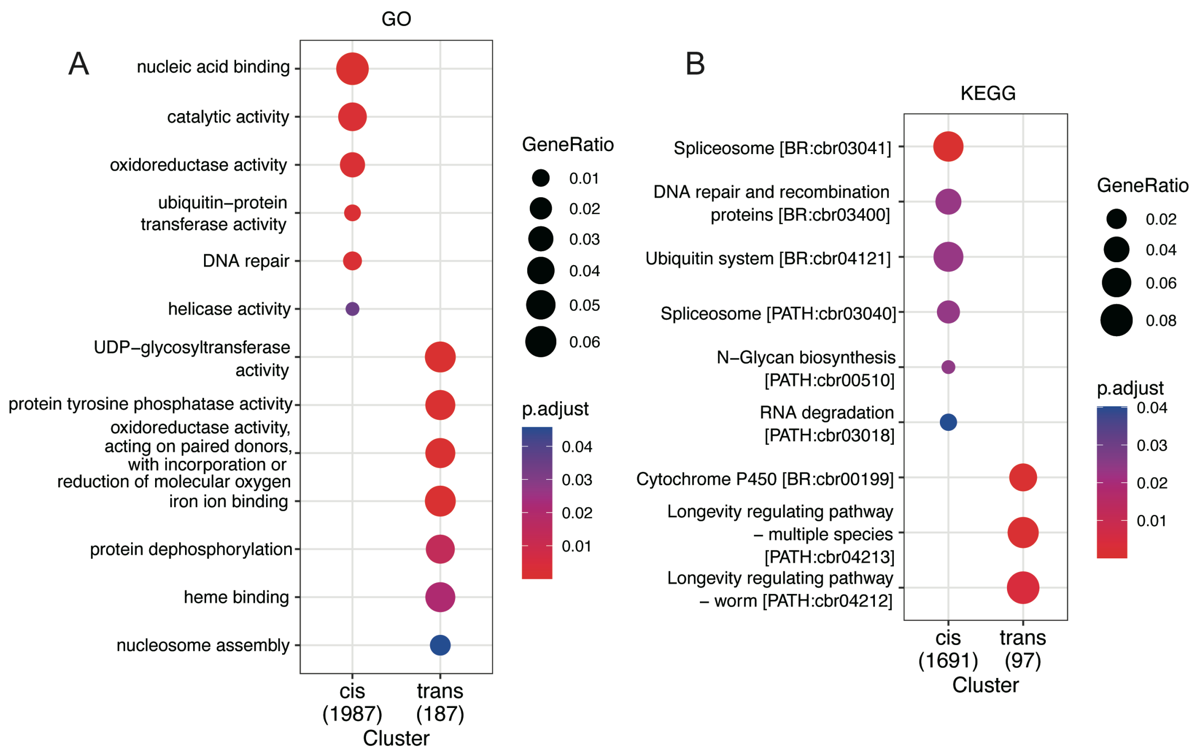

Figure S5: Gene ontology (GO) (A) and Kyoto Encyclopedia of Genes and Genomes (KEGG) (B) pathway enrichment according to regulatory profiles shared by two F1 hybrids.

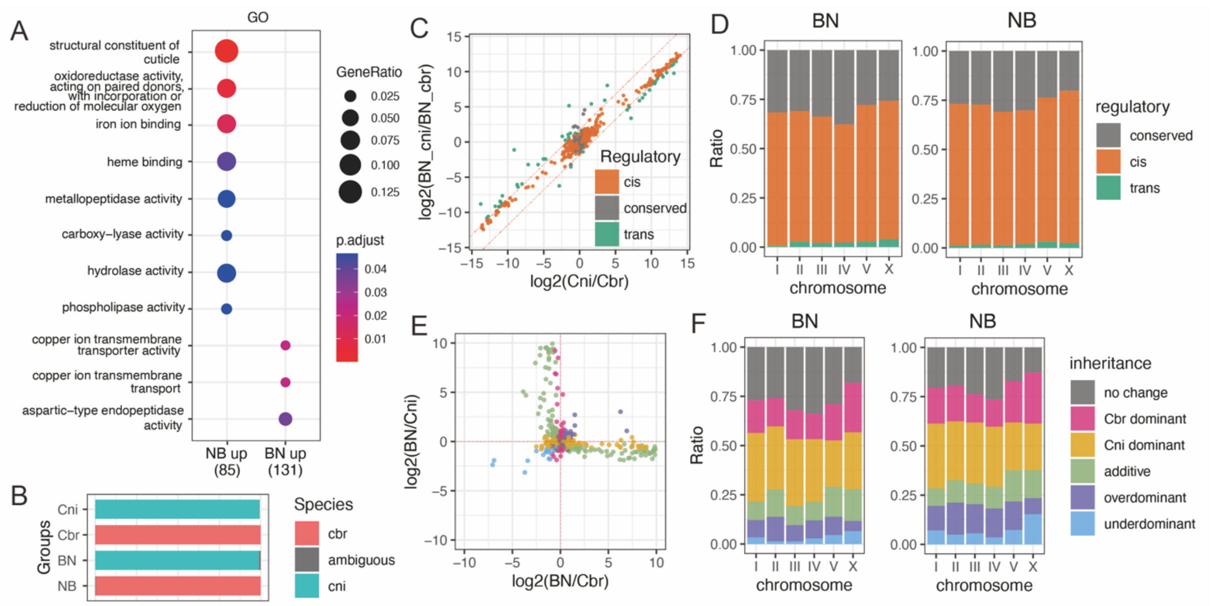

Figure S6: mitochondrial gene analysis. (A) GO enrichment of differential genes between BN and NB, genes that met the condition of Pvalue < 0.05 and |logFC| >1 were used in the enrichment. (B) The mapping ambiguity of the RNA-seq reads against the mixed reference containing both *C. briggsae* and *C. nigoni* mitochondrial genome sequences, there are three replicates in group Cni and two replicates in group Cbr, BN and NB, and here show the average results of each group. (C,D) The *cis*- and *trans*- regulated genes in 902 expressed one-to-n orthologs of mitochondrial related genes. The dot plot showing the method used to define the *cis-, trans-*, or conserved genes of BN (C) and the distribution of *cis, trans* and conserved genes among different chromosomes of F1 hybrids (D). (E, F) Expression inheritance modes between two species in one-to-n orthologs of mitochondria related genes. Dotplot showing the method used to define the one-to-n orthologs in inheritance categories of BN (E) and the distribution of the mitochondria related genes with different inheritance profiles among different chromosomes of F1 hybrids (F).

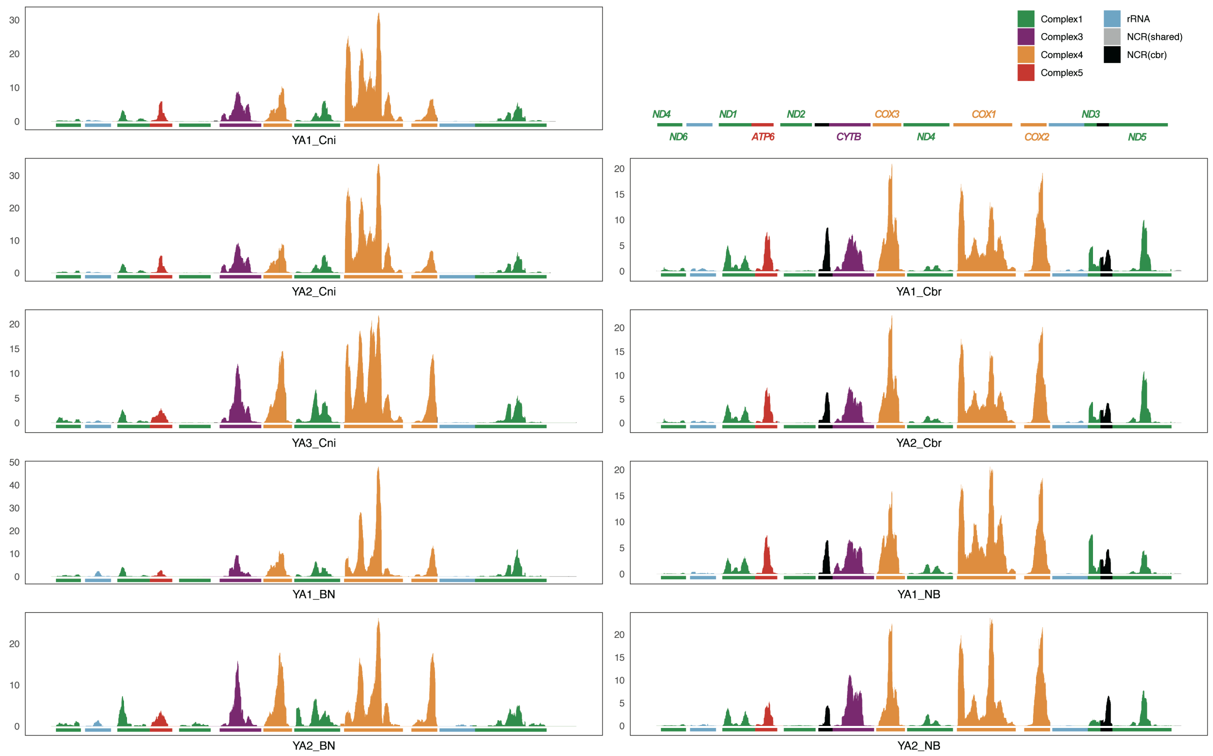

Figure S7: The coverage plots of 12 mitochondria coded genes of 9 groups.

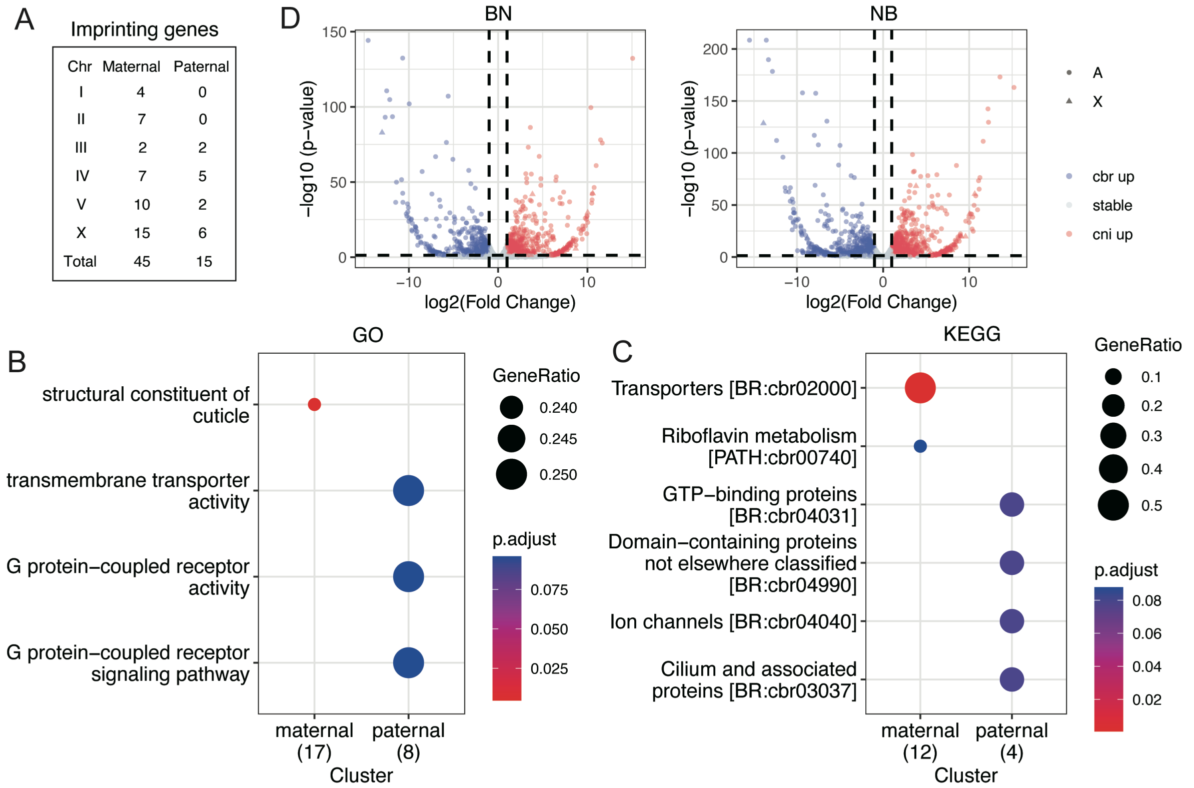

Figure S8: Comparison between two haplotypes from two F1 hybrids. (A) The distribution of all imprinting genes in chromosomes. (B-C) GO (B) and KEGG (C) enrichment of maternal and paternal imprinting genes. (D) Volcano plots showing the differential expressed alleles of BN_cbr and BN_cni, NB_cbr and NB_cni.

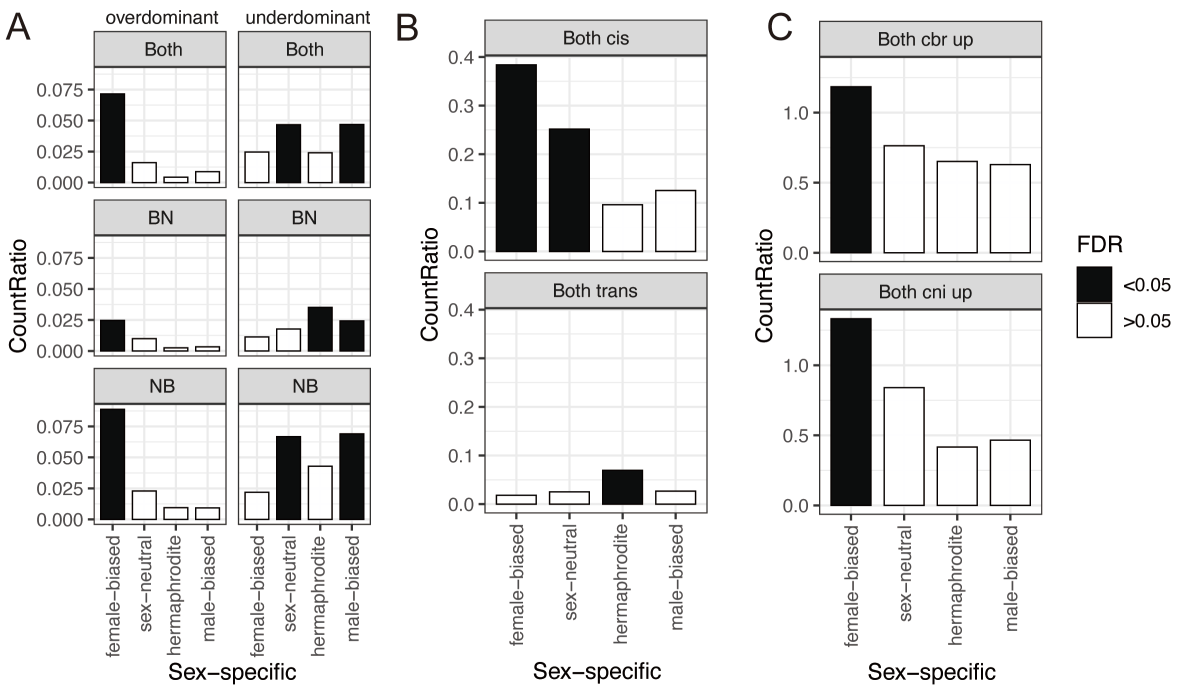

Figure S9: Enrichment of inheritance (A), regulatory (B), and DEG (C) using another recent published sex-specific term (Sanchez-Ramirez, et al. 2021), including female-biased, sex-neural, hermaphrodite and male-biased. All of the significance cut-off is FDR = 0.05. (A) Enrichment analysis against sex-specific gene categories (Fig. 2I) of shared overdominant and underdominant regulated genes in the two F1 hybrids. (B) Enrichment analysis against sex-specific gene categories of shared *cis*- and *trans*-regulated genes (Fig. 3E) in the two F1 hybrids. (C) Enrichment analysis of shared orthologs (Fig. 5G) against the sex-related gene categories.

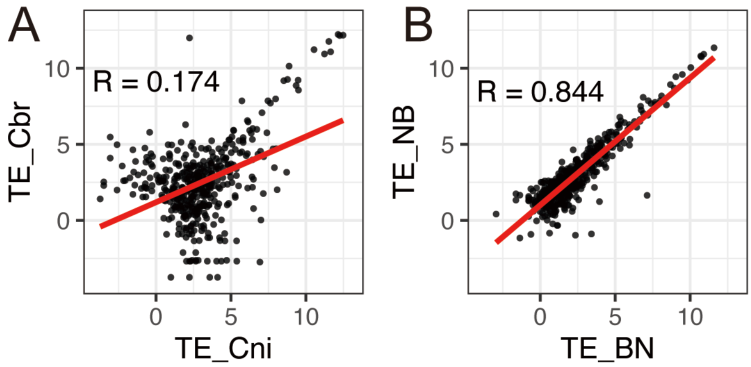

Figure S10: Spearman correlation of all the TEs in the two parent species *C. briggsae* (Cbr) and *C. nigoni* (Cni) (A), and in the two F1 hybrids BN and NB (B).

Reference:

Sanchez-Ramirez S, Weiss JG, Thomas CG, Cutter AD 2021. Widespread misregulation of inter-species hybrid transcriptomes due to sex-specific and sex-chromosome regulatory evolution. PLoS Genet 17: e1009409. doi: 10.1371/journal.pgen.1009409
